# Ampk alpha2 T172 activation dictates exercise performance and energy transduction in skeletal muscle

**DOI:** 10.1126/sciadv.aeb3338

**Published:** 2026-02-25

**Authors:** Ryan N. Montalvo, Xiaolu Li, Gina M. Many, Tyler J. Sagendorf, Qing Yu, Wenqing Shen, Nishikant Wase, A. Robert Burgardt, Tong Zhang, Marina A. Gritsenko, Matthew J. Gaffrey, Hemangi Bhonsle, Yuntian Guan, Xuansong Mao, Mei Zhang, Wei-Jun Qian, Zhen Yan

**Affiliations:** ^1^ Center for Exercise Medicine Research, Fralin Biomedical Research Institute at the Virginia Tech Carilion School of Medicine, Roanoke, VA 24016, USA.; ^2^ Biological Sciences Division, Pacific Northwest National Laboratory, Richland, WA 99354, USA.; ^3^ Center for Skeletal Muscle Research at Robert M. Berne Cardiovascular Research Center, University of Virginia School of Medicine, Charlottesville, VA 22903, USA.; ^4^ PPD, Thermo Fisher Scientific, Richmond, VA, 23229, USA.; ^5^ Department of Human Nutrition, Foods, and Exercise, College of Agriculture and Life Sciences, Virginia Tech, Blacksburg, VA 24061, USA.; ^6^ Division of Cardiovascular Medicine, Department of Medicine, University of Virginia School of Medicine, Charlottesville, VA 22903, USA.

## Abstract

Adenosine 5′-monophosphate–activated protein kinase (AMPK) is an energetic sensor for metabolic regulation and integration. Here, we used CRISPR-Cas9 to generate nonactivatable Ampkα knock-in (KI) mice with mutation of threonine-172 phosphorylation site to alanine (T172A), circumventing the limitations of previous genetic interventions that disrupt the protein stoichiometry. KI mice of Ampkα2, but not Ampkα1, demonstrated phenotypic changes with increased fat-to-lean mass, impaired endurance exercise capacity, and diminished mitochondrial maximal respiration and conductance in skeletal muscle. Integrated temporal multiomics analysis (proteomics/phosphoproteomics/metabolomics) in skeletal muscle at rest and during exercise establishes a pleiotropic yet imperative role of Ampkα2 T172 activation for glycolytic and oxidative metabolism, mitochondrial respiration, and contractile function. There is a substantial overlap of skeletal muscle proteomic changes in Ampkα2 T172A KI mice with that of patients with type 2 diabetes. Our findings suggest that Ampkα2 T172 activation is critical for exercise performance and energy transduction in skeletal muscle and may serve as a therapeutic target for type 2 diabetes.

## Teaser

Integrative phenotypic and multiomic analysis reveals distinct AMPKα2 T172 regulatory clusters driving muscle energy metabolism.

## INTRODUCTION

Adenosine 5′-monophosphate (AMP)–activated protein kinase (AMPK) is a critical energy-sensing molecule within skeletal muscle that has several established roles in regulating glucose uptake, fatty acid oxidation, and overall metabolic plasticity at rest, during exercise, and in response to exercise training (*1*–*5*). AMPK exists as a heterotrimeric protein complex composed of a catalytic α subunit (α1 or α2 isoforms), a scaffolding β subunit (β1 or β2 isoforms), and a regulatory γ subunit (γ1, γ2, or γ3 isoforms) and is stimulated by AMP and/or adenosine diphosphate (ADP) binding to the γ subunit, resulting in a conformational change that exposes the catalytic α threonine-172 (T172) site for phosphorylation by upstream kinases (e.g., CaMKKb and LKB1) (*6*–*8*), leading to full activation. In this regard, AMPK functions as a bellwether of cellular energy status in skeletal muscle, providing a mechanism by which the bioenergetic demand of exercise can trigger an immediate response to enhance metabolic flux during exercise and induce adaptive modifications that improve functional capacity for future endeavors.

Genetic manipulations of Ampk through global or muscle-specific deletion of the Ampk α1/α2, β1/β2, or γ3 gene or forced expression of kinase-dead mutant Ampk in mice have demonstrated Ampk’s function in the regulation of glucose uptake, oxidative metabolism, mitochondrial biogenesis and respiration, and exercise performance (*5*, *9*–*32*). Further, Ampk has also been shown to regulate mitophagy and mitochondrial quality control (QC) in skeletal muscle in response to exercise training (*27*, *33*). Several reports with genetic ablation of the Ampk genes demonstrated that Ampkα2 has a greater role than Ampkα1 in regulating skeletal muscle metabolic function, given that Ampkα2 is the dominant isoform in skeletal muscle (*25*, *29*, *31*). Several of these studies advocate for the necessity of Ampkα2 signaling for the metabolic regulation in skeletal muscle (*17*, *18*, *20*, *23*), whereas others contend that Ampkα2 is dispensable for enhanced fatty acid oxidation induced by exercise or muscle contraction (*19*, *34*). These contradicting findings warrant further clarification on these key determinants of skeletal muscle function.

Deletion of a gene may not only disrupt protein stoichiometry but also cause ill-defined off-target effects. These unintended changes may cause compensatory adaptations, leading to spurious interpretation of the outcome measures (*35*, *36*). Previous combinations of deletion of the Ampkα1/2, β1/2, or γ1/2/3 genes all caused disruption protein stoichiometry in Ampk holoenzyme and its protein complexes (*30*, *37*–*39*). Therefore, despite the valuable insights provided by these genetic models, many of the phenotypic changes observed are discreet, and the interpretations may be confounded (*40*).

Here, we took advantage of CRISPR-Cas9–mediated gene editing to substitute the T172 phosphorylation site for alanine (T172A) in the α1 and α2 subunits separately, creating two global nonactivatable knock-in (KI) models, circumventing the limitations of previous genetic approaches by maintaining protein stoichiometry. This approach made it possible to definitively ascertain the role of Ampk activation via phosphorylation in regulation of muscle contractile and metabolic function and exercise-induced adaptations. We performed comprehensive biochemical, metabolic, and physiological assessments to dissect the functional role(s) of Ampkα1 and Ampkα2 activation on mitochondrial bioenergetics, metabolic control, and exercise performance. Further, we used an integrated multiomic (proteomic, phosphoproteomic, and metabolomic) approach to identify the impacts of ablated Ampkα2 activation on functional and metabolic phenotypes in skeletal muscle at rest and during exercise. Our findings provide robust evidence that phosphorylation of Ampkα2 at T172 is imperative to the maintenance of metabolic energy transfer and bioenergetic machinery at baseline and the regulation of these pathways during exercise in skeletal muscle. These results lay a detailed road map for directing research toward pharmacological and behavioral therapies that target Ampk for treating chronic metabolic disease, such as type 2 diabetes (T2D).

## RESULTS

### Ampkα2 T172A KI mice manifest an altered metabolic phenotype and exercise capacity

**Fig. 1.**
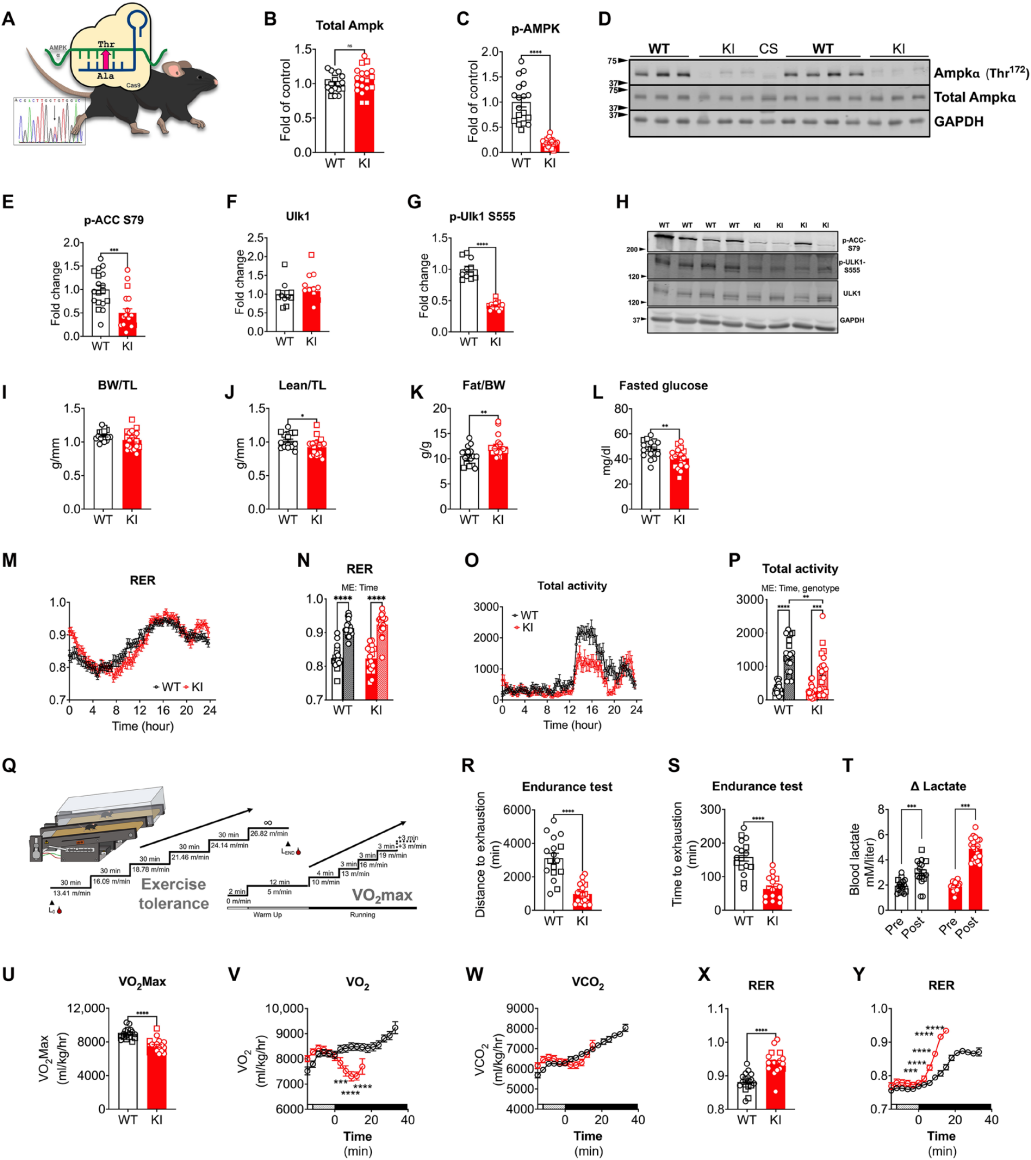
Ampkα2 KI mice demonstrate altered metabolic regulation and limited exercise capacity. Ampkα2 T712A KI and WT littermate mice were tested for genotype and phenotypic changes in body composition, metabolic function, and exercise capacity. (**A** to **D**) Genotype was confirmed by DNA sequencing and Western blot. WT, wild type; KI, Ampkα2 T172A KI; CS, common standard. (**E** to **H**) Western blot confirmation of classical Ampk signaling targets pACC (phosphorylated acetyl–coenzyme A carboxylase) and total and pS555-Ulk1 and representative image. (**I** to **L**) Body composition analysis (EchoMRI) for lean mass (J) normalized to tibia length (TL, millimeters) and fat mass (K) normalized to body weight (BW, grams). (L) Blood glucose measurement was taken after an overnight fast (1700 to 0900). (**M** to **P**) Metabolic cage measurements (Columbus Instruments) were taken over 24 hours (hr) for RER and total activity. Comparisons made between the light/resting (0700 to 1900; time 0 to 12 hours) and dark/active (1900 to 0700; time 12 to 24 hours) cycles. (**Q**) Illustration of protocols for endurance treadmill running and VO_2_Max tests for evaluation of exercise capacity. Blood collection for lactate before beginning (L_0_) and at the end (L_END_) was indicated. (**R** to **T**) Endurance testing evaluated distance (meters) and time (minutes) and confirmed exhaustion through pre-post blood lactate measurements. (**U** to **Y**) Gas exchange measurements were performed during VO_2_Max test for VO_2_Max, VCO_2_, and RER. Males (*n* = 6 WT; *n* = 7 KI) represented in squares and females (*n* = 11 WT; *n* = 12 KI) in circles for all outcomes; circles represent genotype average for (N), (P), (V), (W), and (Y). Data presented as means ± SEM. Statistical analysis performed by *t* test between groups. Two-way analysis of variance performed for (N) and (P) for main effects (ME) of time and genotype. Significance indicated as *P < 0.05, **P < 0.01, ***P < 0.001, and ****P < 0.0001; ns, not significant.

We first validated successful CRISPR-Cas/9 Ampkα T172A substitution (KI) by DNA sequencing and Western blot (Fig. 1, A to D, and fig. S2, A and B). In addition to reduced Ampk phosphorylation, skeletal muscles from Ampkα2 KI mice showed decreased phosphorylation of classical Ampk targets (*41*, *42*) (e.g., p-Acc; p-Ulk1), further confirming the validity of the KI model (Fig. 1, E to H); additional verification was also performed in gastrocnemius muscle, heart, and liver that confirmed the KI genotype (fig. S1, A to D). We then sought to extensively characterize the metabolic phenotypes of these KI mice. First, body composition analysis by echoMRI revealed slightly reduced lean mass and increased fat mass in Ampkα2 KI mice (Fig. 1, I to K). Subsequently, we performed glucose tolerance and insulin tolerance tests. The Ampkα2 KI mice showed slightly improved glucose clearance [area under the curve (AUC)] and fasting glucose with no change in insulin tolerance (Fig. 1l and fig. S1, E to H). Further, plantaris muscle weight and heart weight were lower in α2 KI mice without differences in gastrocnemius or soleus muscle weights (fig. S1, I to L). Mice were then placed in metabolic cages to evaluate whole-body metabolism over 24 hours. Both wild-type (WT) and Ampkα2 KI mice showed an increased respiratory exchange ratio (RER) in the dark cycle with normal circadian patterns, indicating preserved metabolic activity. However, total and ambulatory activity were significantly lower in Ampkα2 KI mice in the dark cycle (Fig. 1, M to P, and fig. S1, M and N), suggesting underlying impairments in muscle function. No significant differences were observed for Ampkα1 KI mice related to any of these outcome measures, suggesting minimal functional roles of Ampkα1 activation in skeletal muscle (fig. S2, C to P).

Various genetic deletions of Ampk genes in skeletal muscle demonstrate negative impacts on exercise capacity (*5*, *16*, *18*, *43*, *44*), yet disrupted protein stoichiometry may have convoluted these results. To this end, we measured exercise performance for Ampkα1 KI and Ampkα2 KI mice in treadmill running endurance test as well as a separate VO_2_max test to investigate the role of Ampk activation during exercise. The procedures for each exercise test are illustrated in Fig. 1Q. Ampkα2 KI mice had dramatically diminished time to fatigue during the endurance test compared to WT littermate mice (Fig. 1, R to T); this effect was not observed in Ampkα1 KI mice (fig. S2, Q to S). Further, VO_2_max was significantly diminished in Ampkα2 KI mice (Fig. 1, U to Y). These results are further corroborated by impaired metabolic regulation represented by an early elevation of RER, which suggests aberrant fatty acid oxidation and a lower anaerobic threshold (Fig. 1, X and Y, and fig. S1, O to Q). Like the endurance test, no significant effects on VO_2_max outcomes were observed for the Ampkα1 KI mice (fig. S2, T to V). Together, these results demonstrate a deleterious effect of Ampkα2 T172 signaling ablation within the skeletal muscle, as is evident by significantly reduced functional capacity as well as impaired metabolic regulation.

### Global proteomics demonstrates prevalent regulatory role of Ampkα2 T172 activation in mitochondrial bioenergetic machinery

Our data demonstrate that Ampkα2 T172 activation dictates the gross functional capacity and metabolic signature in skeletal muscle at rest and during exercise, which is consistent with previous studies (*2*, *5*); however, the molecular mechanisms underlying the role of Ampkα2 T172 activation has not been investigated. We thus conducted a global proteomic analysis of Ampkα2 KI skeletal muscle with two objectives: (i) to provide high-resolution mapping of the known roles of Ampkα2 in various regulatory processes, and (ii) to identify novel targets of Ampkα2 activation that may affect exercise and skeletal muscle functional capacity.

**Fig. 2.**
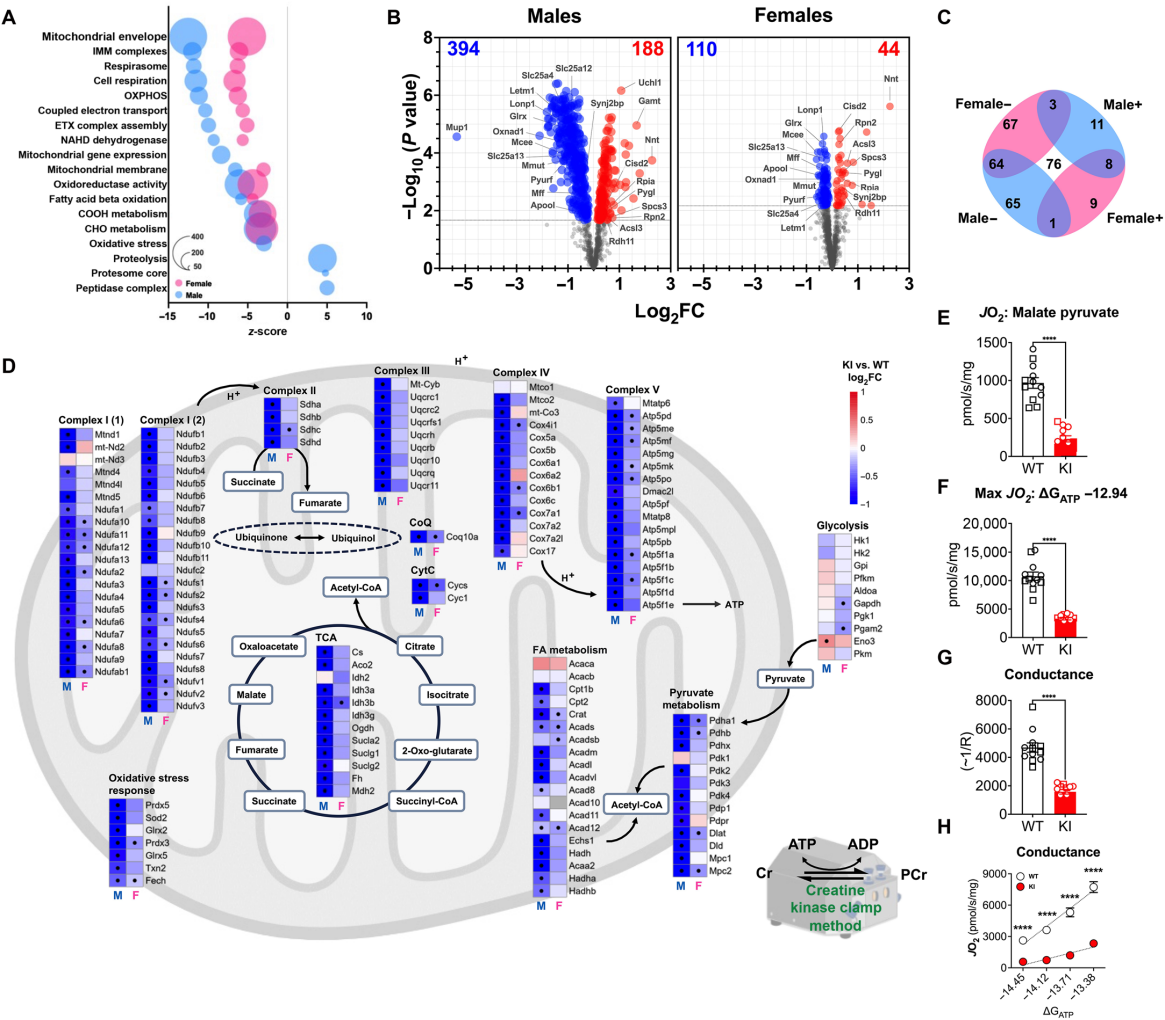
Global proteomic analysis reveals Ampkα2 KI mice have disrupted core metabolic machinery for mitochondrial function. Global proteomics was performed for gastrocnemius muscle from male and female WT (*n* = 6) and Ampkα2 KI (*n* = 6) mice. (**A**) Dot plot of the most significantly regulated GO terms (GO biological processes, cellular components, and molecular function represented by *z* score (*x* axis), number of proteins per GO term (size of dot), separated by males (blue) and females (pink) as indicated in legend. (**B**) Volcano plot of proteins represented in males [394 proteins significantly down-regulated (blue) and 188 up-regulated (red)] and females (110 down-regulated and 44 up-regulated) limited to include GO term gene sets designated in (A). (**C**) Four-way representation of significantly regulated proteins that overlapped between males and female volcano plots; up-regulated (+) and down-regulated (−), 76 proteins in total (center). (**D**) Enrichment analysis of proteins related to glycolysis and mitochondrial oxidative metabolism [pyruvate metabolism, TCA, fatty acid (FA) oxidation, oxidative stress response and electron transport chain complexes (I, II, III, IV, and V)]; • representative of adjusted *P* < 0.05 difference within heatmap by *t* test. (**E** to **H**) Creatine kinase clamp analysis of mitochondrial respiration with malate-pyruvate substrates in a non-phosphorylating substrate–stimulated state and maximal respiration (MAX *J*O_2_; ∆*G*_ATP_ −12.94). Conductance measured through PCr titrations of 1 mM (∆*G*_ATP_ −13.38 kCal/mol), 2 mM (∆*G*_ATP_ −13.71 kCal/mol), 4 mM (∆*G*_ATP_ −14.12 kCal/mol), and 6 mM (∆*G*_ATP_ −14.45 kCal/mol) as slope of ∆*G*_ATP_ and *J*O_2_. Males (*n* = 6 WT; *n* = 6 KI) are represented in squares and females in circles (*n* = 6 WT; *n* = 6 KI); average of genotypes represented in circles in (H). Data presented as means ± SEM. Statistical analysis performed by *t* test between groups: ****P < 0.0001. IMM, inner mitochondrial membrane.

Relative to WT mice, Ampkα2 KI mice gastrocnemius muscle displayed 444 significantly altered proteins in females and 1310 in males (adjusted *P* < 0.05) (data file S1). We next applied the preranked version of the correlation adjusted mean rank (CAMERA-PR) gene set test (*45*) to identify concordant changes in Gene Ontology (GO) terms (molecular function, biological processes, and cellular components) (fig. S3, A to C). Ampkα2 KI mice had substantive changes of proteins in mitochondrial and core metabolic pathways that include the electron transport chain, cellular respiration, tricarboxylic acid (TCA) cycle, and adenosine triphosphate (ATP) metabolic process (Fig. 2, A and B). Of these, male and female KI mice shared 76 differentially expressed proteins, 64 of which decreased in abundance (Fig. 2C). Further, Ampkα2 KI mice showed significantly decreased electron transport and core metabolic proteins in skeletal muscle in both sexes (Fig. 2D), although Ampkα2 KI male mice had a greater impact on mitochondrial proteins compared to females. Pyruvate dehydrogenase (Pdh), which serves as an integration point between anaerobic and aerobic pathways, was decreased in both male and female Ampkα2 KI mice (Fig. 2D). Male but not female α2 KI mice displayed reduced abundance of proteins related to pyruvate transport (Mpc1/Mpc2), and the Pdh subunits E1 [Pdha1, Pdhb, Pdhx, E2 (Dlat), and E3 (Dld)] as well as Pdh phosphatase (activating) (Pdpr; Pdp1) and Pdh kinase (inactivating) isozymes 2 and 4 (Pdk2 and Pdk4). Pdh E3 simultaneously produces reduced form oxidized nicotinamide adenine dinucleotide [(NAD^+^) NADH] and FADH_2_, the substrates of electron transport chain (ETC) complex I and II, respectively, demonstrating a dramatic effect of Ampkα2 T172 inactivation on Pdh activation in male mice. Male mice appear to be more susceptible to the disruption of this signaling pathway. These findings suggest that Ampkα2 activation in skeletal muscle plays a fundamentally important role in maintaining the metabolic machinery.

To gain further insights into the regulatory role of Ampkα2 T172 in skeletal muscle mitochondrial processes, we performed gene set enrichment analysis using the reference gene set MitoCarta3.0 (*46*) to identify mitochondria-specific alterations in protein abundance. This investigation revealed decrements of proteins related to mitochondrial genetic replication (i.e., TOM, TIM, RNA granule, and ribosomes), mitochondrial and ETC assembly and integrity [i.e., MICOS and mitochondrial isolation buffer (MIB) complexes], and mitochondrial QC (e.g., fusion, fission, and mitophagy) (fig. S4, A to D). Again, the decrements were more severe in male KI mice compared with female KI mice.

In an effort to directly dissect the role of Ampkα2 T172 activation on mitochondrial function, we measured mitochondrial respiration in isolated mitochondria from gastrocnemius, matching global proteomics analysis. Further, we use a creatine-kinase clamp protocol amended to include phosphocreatine titrations as a measure of mitochondrial conductance over a range of respiratory demands (∆*G*_ATP_) (detailed in fig. S4, A and F) (*47*, *48*). Substrate-stimulated nonphosphorylating respiration using malate pyruvate and phosphorylating maximal respiration (MAX *J*O_2_; ∆*G*_ATP_ −12.94) were greatly decreased in Ampkα2 KI mice (Fig. 2, E and F). Measurement of conductance (slope of *J*O_2_ versus ∆*G*_ATP_ −13.38, −13.71, −14.12, and −14.45) mimics a tractable bioenergetic stress test, and Ampkα2 KI mice demonstrated increased resistance within the electron transport chain and impaired ability to respond to energetic demand (Fig. 2, G and H). The roles of Ampk on mitochondrial quality and function have been implicated in several studies (*5*, *12*, *27*, *33*, *38*), yet this report shows direct evidence of the essential role of Ampkα2 T172 activation in maintaining normal mitochondrial machinery, respiration, and conductance in skeletal muscle.

### Exercise reveals a potential role of Ampkα2 T172 activation in skeletal muscle metabolic and contractile function via phosphoproteome regulation

**Fig. 3.**
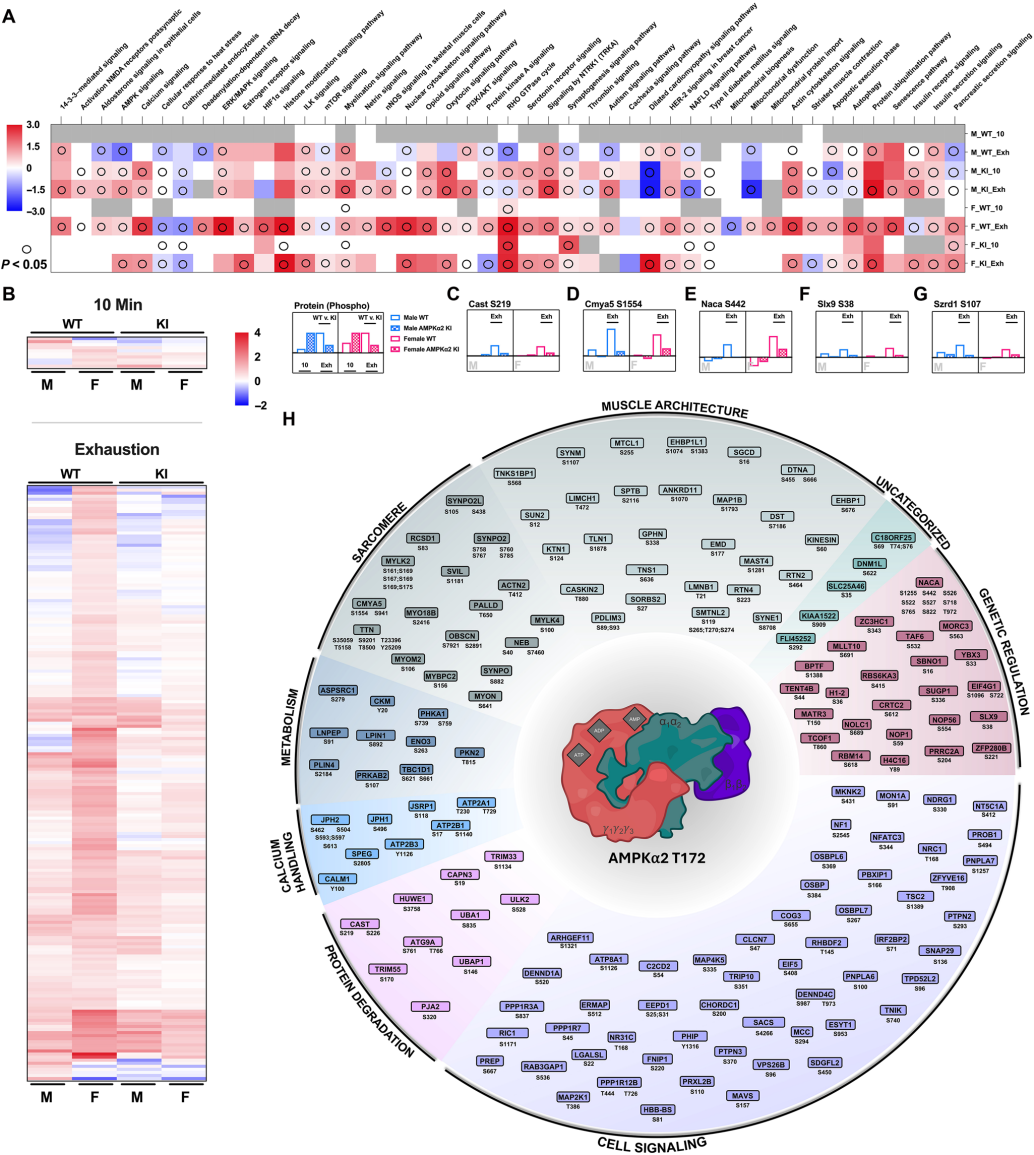
Ampkα2 T712 activation dictates the phosphoproteomic response to exercise in skeletal muscle. Male and female Ampkα2 KI and WT littermate mice were divided into three groups (i) sedentary, (ii) exercise for 10 min (10 min) and (iii) exercise to exhaustion (Exh) groups. Protocol detailed in fig. S5. (**A**) Ingenuity Pathway Analysis heatmap of phosphorylation sites comparing male (M) and female (F) at 10 min (10) and Exh. Empty circle O indicates the significance of the pathway with *P* < 0.05. (**B**) Putative phosphorylation sites significantly activated (adjusted *P* < 0.05) by exercise but not activated in male female KI mice at 10 min (top) and Exh (bottom). Data organized by hierarchical clustering: Euclidean complete. (**C** to **G**) Log_2_FC of phosphorylation sites representing significantly increased phosphorylation in male (blue bar) and female (pink bar) Ampkα2 KI (checkered bar) and WT (open bar) mice. A significant decrease of phosphorylation was observed in Ampkα2 KI (*P* < 0.05) at Exh for Calpastatin (*Cast*) S219, cardiomyopathy associated protein 5 (*Cmya5*) S1554, nascent polypeptide associated complex subunit alpha (*Naca*) S442, SLX9 ribosome biogenesis factor (*Slx9*) S38, and SUZ RNA binding domain containing 1 (*Szrd1*) S107. Bars above time points indicate significant differences between WT and Ampkα2 KI mice at 10 min or Exh. (**H**) All potential sites identified as under regulation by Ampkα2 activation at 10 min or Exh designated into functional groups irrespective of sex.

Here, we used phosphoproteomics to distinguish skeletal muscle signaling in response to treadmill running to identify previously unknown targets of Ampk that may affect overall functional capacity and metabolic plasticity. On the basis of the early elevation of RER during VO_2_max testing, we collected samples at an intermediate 10-min time point and at exhaustion (EXH) to compare exercise responses to the sedentary states (protocol demonstrated in fig. S5A and data file S2). The phosphoproteomic response at 10 min was significantly greater in Ampkα2 KI mice compared with WT mice, which is consistent with the observations of reduced exercise capacity in Ampkα2 KI mice (fig. S5, B and C). At EXH, male Ampkα2 KI mice had more changes compared to WT mice. Female WT mice showed more changes than female Ampkα2 KI at EXH. Overall, female mice had significantly fewer changes compared to males, displaying a clear sex difference. To elucidate the functionally diverse phosphoproteomic response to acute exercise, we performed ingenuity pathway analysis, which revealed a variety of signaling mechanisms affected by Ampkα2 T172 signaling ablation, including those related to insulin signaling, extracellular signal–regulated kinase/mitogen-activated protein kinase, mammalian target of rapamycin (mTOR), muscle structure and contraction, neuronal nitric oxide synthase (nNOS), apoptosis, and actin cytoskeleton (Fig. 3A and data file S3).

The main goal of this analysis is to identify putative substrates of activated Ampkα2 via T172 phosphorylation by exercise. To this end, we compared fold change of phosphorylation at various phosphorylate sites in relationship to the sedentary condition for both genotypes separately at the 10-min time point and at EXH with results limited to those with adjusted *P* < 0.05. Further, we compared WT with Ampkα2 KI mice to observe significant differences at these separate time points (i.e., ∆log_2_FC; data file S2) with consideration of sex. This analysis revealed significant changes of phosphorylation at 204 sites with 10 unique phosphorylation sites that were significantly regulated at the 10-min time point and 194 at EXH (Fig. 3B and fig. S6). Only five sites were significantly increased at EXH in WT mice that were also significantly diminished in Ampkα2 KI for both the males and females: calpastatin (Cast) S219, cardiomyopathy-associated protein 5 [Cmya5, (myospryn)] S1554, nascent polypeptide–associated complex subunit alpha (Naca) S442, SLX9 ribosome biogenesis factor (Slx9) S38, and SUZ RNA binding domain containing 1 (Szrd1) S107 (Fig. 3, C to G). We consider these novel targets of Ampkα2.

Further evaluation of the significantly regulated phosphorylation sites allowed for binning of genes into eight physiologically relevant groups (Fig. 3H): muscle architecture, sarcomere, metabolism, genetic regulation, general cell signaling, protein degradation, calcium handling, and uncategorized cellular processes. Although the specific role for many of these phosphorylation sites have yet to be determined, these genes indicate a significant regulatory control of Ampkα2 activation via T172 phosphorylation on the functional process of excitation contraction coupling, muscle structural stability, and contractile capacity and metabolism (fig. S6). First, calcium handling at T-tubule ryanodine receptor (e.g., Junctophillin 1/2 (Jph1/Jph2), sarcoplasmic reticulum calcium ATPase 1 (Atp2a1), and intracellularly (e.g., calmodulin) are significantly altered. Additional changes were noted in sarcomere and specifically at Z disk [e.g., Cmya5; synaptotodin (Synpo); supervilin (Svil); Titin (Titin)], which is complemented by alterations in exercise-responsive phosphorylation sites related to gross muscle architecture [e.g., sarcoglycan delta and dystonin (Dst)]. Changes in sarcomeric protein phosphorylation are consistent with global proteomics results, suggesting increased phosphorylation of proteins related to protein degradation in response to exercise via the ubiquitin proteasome system [e.g., Cast; calpain-3 (Capn3)]. Of note, our results substantiate recent reports that identified C18orf25 (*49*) and Fnip1 (*50*) as Ampk targets and essential regulators of exercise capacity.

The role of Ampkα2 activation in metabolism is well-demonstrated in the current analysis, primarily in glucose regulation. We observed significant phosphorylation of Ampk upstream regulator, serine/threonine–protein kinase N2 (Pkn2), which has been shown to considerably affect glucose metabolism through Ampk activation in skeletal muscle (*51*). Glycolytic regulation and glucose handling are also highlighted with significant results in Tbc1d1 and enolase (Eno3) as well as glycogen metabolism via phosphorylase kinase alpha. Further cytosolic muscle–specific creatine kinase (Ckm Y20) was phosphorylated at the 10-min and exhaustive time points for male WT mice but not in the Ampkα2 T172A KI mice. Ckm is essential for the metabolic response to exercise and metabolic flexibility within skeletal muscle, providing a novel and substantial regulatory role of Ampkα2 T172 activation.

In an effort to identify the direct targets of Ampk phosphorylation, we used the 204 unique phosphorylation sites that showed significant changes in Fig. 3B to perform alignment analysis for the Ampk consensus motif using a validated motif matrix and pipeline (*52*). These analyses produced strict matches (*n* = 7), relaxed matches (*n* = 18), and minimal matches (*n* = 66) (Table 1 and data file S4). This finding implicates a potentially significant role of Ampkα2 T172 in regulating phosphatase Ppp1r12b S446 during exercise. Several metabolic regulators are also noted, including Crtc2 S612, Ulk2 S528, and Ppp1r3a S837. Last, the importance of Ampk signaling on the sarcomere (Mylk4 S100) and muscle integrity (Dst S7186, Smtn12 S119, and Sorbs2 S27) is highlighted in this analysis.

**Table 1.** Phosphoproteomics mapping of AMPK consensus motif. Proteins listed in alphabetical order that had a strict or relaxed fit type for the consensus motif of AMPK. Amino acid nomenclature according to standard naming practices. Sequence position mapped based on amino acid center (S) −5 of center, −3 of center, and +4 of center. The pattern for strict match was [I/V/L/M] - X - [R/K] - X - X - [S/T] - X - X - X - [I/F/L/M], in which the −5 position must be I, V, L, or M, −3 must be R or K, and +4 must be I, F, L, or M. Relaxed matching pattern and full results available in data file S4.

				Amino acid position				Match type	
#	Protein	Site	Sequence	−5	−3	0	+4	Strict	Relaxed
1	Ankrd11	S1070	HSKDRKAsFDQLREK	K	R	S	L	FALSE	TRUE
2	C2cd2	S54	DELRRREsDTLLSWI	L	R	S	L	TRUE	TRUE
3	Cog3	S655	PRFFRLNsNNALIEF	F	R	S	L	FALSE	TRUE
4	Crtc2	S612	THCSRHGsGPNIILT	C	R	S	I	FALSE	TRUE
5	Dst	S7186	SKMLRSEsNSSITAT	M	R	S	I	TRUE	TRUE
6	Ehbp1l1	S1074	LGVQKPGsWGALKYE	V	K	S	L	TRUE	TRUE
7	Map4k5	S335	SRAERTAsEINFDKL	A	R	S	F	FALSE	TRUE
8	Mylk4	S100	MVMAKHAsVDNLYTV	M	K	S	L	TRUE	TRUE
9	Ppp1r12b	S446	LGLRKTGsHNMLSEV	L	K	S	L	TRUE	TRUE
10	Ppp1r3a	S837	TGNQKATsKLDLHLG	N	K	S	L	FALSE	TRUE
11	Prep	S667	VGRSRKQsNPLLIHV	R	R	S	L	FALSE	TRUE
12	Prob1	S494	VGLSRDSsLPALLPR	L	R	S	L	TRUE	TRUE
13	Prxl2b	S110	LGFKRYNsLSILPAA	F	R	S	L	FALSE	TRUE
14	Ptpn2	S293	QKRWKELsKEDLSPI	R	K	S	L	FALSE	TRUE
15	Rbm14	S618	LSDYRRLsESQLSFR	D	R	S	L	FALSE	TRUE
16	Smtnl2	S119	LGTARFSsHATFSLS	T	R	S	F	FALSE	TRUE
17	Sorbs2	S27	TSVKRVQsSPNLLAA	V	R	S	L	TRUE	TRUE
18	Ulk2	S528	LLGARLQsAPTLTDI	G	R	S	L	FALSE	TRUE

Further experiments will be required to elucidate Ampk’s integrative regulation of skeletal muscle architecture, contractile capacity, and energetic supply. Nevertheless, the current study provides evidence that Ampkα2 T172 activation synchronizes skeletal muscle metabolic function and contractile capacity through coordinated regulation metabolic and contractile mechanisms during exercise.

### Ampkα2 T172 activation dictates glycolytic and oxidative flux during exercise

**Fig. 4.**
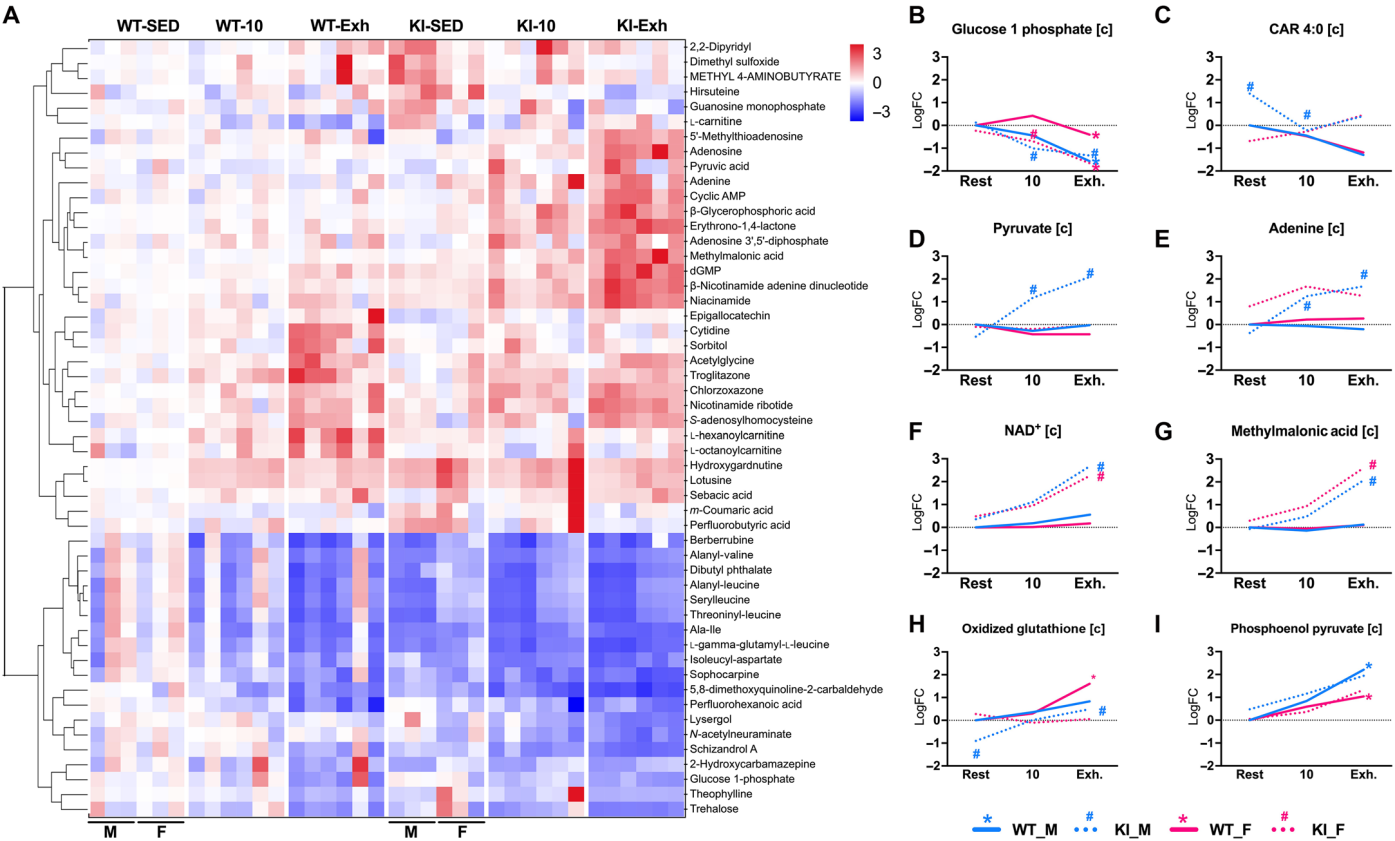
Metabolomic response to exercise demonstrates increased reliance on glycolysis in Ampkα2 KI mice. Metabolomic analysis was performed (protocol detailed fig. S5) for gastrocnemius muscle for Ampkα2 KI and WT mice (WT, *n* = 6; KI, *n* = 6) in sedentary state (SED), at 10 min of (10) exercise and at Exh. (**A**) Heatmap of data from male (M) and female (F) mice; organized by Euclidean complete hierarchical clustering (*P* < 0.01), individual metabolites listed to right of heatmap. (**B** to **I**) Representative tracings of male (blue) and female (pink) WT (solid line) and Ampkα2 KI (dotted line) metabolites during the exhaustive exercise protocol. Adjusted *P* < 0.05 indicated by colored * (WT) or ^#^ (KI) at rest (0), 10 min (10), and Exh.

We then performed a temporal metabolomic analysis to help reconcile our phosphoproteomic and proteomic findings and ascertain metabolic regulation in Ampkα2 KI mice (Fig. 4A, fig. S5, and data file S5). At 10 min, an irregular response in the metabolites relating to glucose 1 phosphate (G1P; Fig. 4B), trehalose, l-aspartic acid, and fatty acid metabolism (e.g., l-carnitine; Fig. 4C) was observed in male Ampkα2 KI mice. Decreased levels of metabolites indicates a more rapid use and reliance on these pathways for energy production in the absence of Ampkα2 T172 activation. Contrastingly, at 10 min, some metabolites increased in abundance in male KI mice, which included adenine (Fig. 4D), ADP, and pyruvate (Fig. 4E), indicating decreased utilization of these substrates. These metabolites and NAD^+^ (Fig. 4F) were also increased at EXH in Ampkα2 KI mice, demonstrating a more wide-spread impairment of these metabolic pathways. Of note, 4-hydroxy-l-proline, l-kynurenine, and methylmalonic acid (Fig. 4G) were significantly increased at EXH in Ampkα2 KI but not WT mice. WT mice but not Ampkα2 KI mice showed significantly reduced propionic acid level at EXH, highlighting the importance of Ampkα2 activation in anaplerotic metabolism. Male KI mice further demonstrated a heightened oxidative environment with increased level of oxidized glutathione at EXH (Fig. 4H), which may potentially impair metabolic flux and contribute to sarcomere instability.

WT and Ampkα2 KI mice show decreased G1P at EXH, while male KI mice show this change at 10 min of exercise. A similar pattern was also observed for carbohydrate trehalose. These findings suggest a more severe dysregulation of glycolytic metabolism in male Ampkα2 KI mice with exercise. Phosphoenolpyruvic acid (Fig. 4I) was increased in both WT (adjusted *P* < 0.05) and Ampkα2 KI mice (adjusted *P* = 0.10 M, 0.09 F) at EXH, along with increased pyruvate at 10 and 90 min of exercise for male KI mice. These findings are line with the observed increase of Eno3 expression, suggesting increased glycoltyic flux for male Ampkα2 KI mice (Fig. 2D). Last, WT mice demonstrate a decrease in lactic acid without a significant change in Ampkα2 KI mice. Agreement among sex is present in many of these results but the accumulation of pyruvate, adenine, and oxidized glutathione in male Ampkα2 KI mice at EXH suggest a sex-specific aberrant impact. Our integrative analysis highlights that Pdh kinase 2 and 4 (inactivating) are positively regulated by increased ATP:ADP and NADH:NAD^+^, which were diminished in WT but not Ampkα2 KI mice during exercise (Figs. 2D and 4, E and F). These results suggest that Ampkα2 KI mice initiate glycolysis more rapidly and are more reliant on this process during exercise, contrasted by a decreased flux through the TCA cycle. This notion is in line with our VO_2_max testing, mitochondrial respiratory outcomes, and phosphoproteomics analysis.

### Significant overlap of skeletal muscle proteomic features between Ampkα2 KI mice and human patients with diabetes

Ampk could be an important target for mitigating obesogenic pathology in skeletal muscle, but not enough is understood about the role of Ampkα2 T172 activation in the context of T2D (*53*). To this end, we investigated the overlap of the current dataset with that of skeletal muscles of patients with diabetes to identify specific targets of Ampkα2 T172 activation that may serve to mitigate diabetic pathology.

**Fig. 5.**
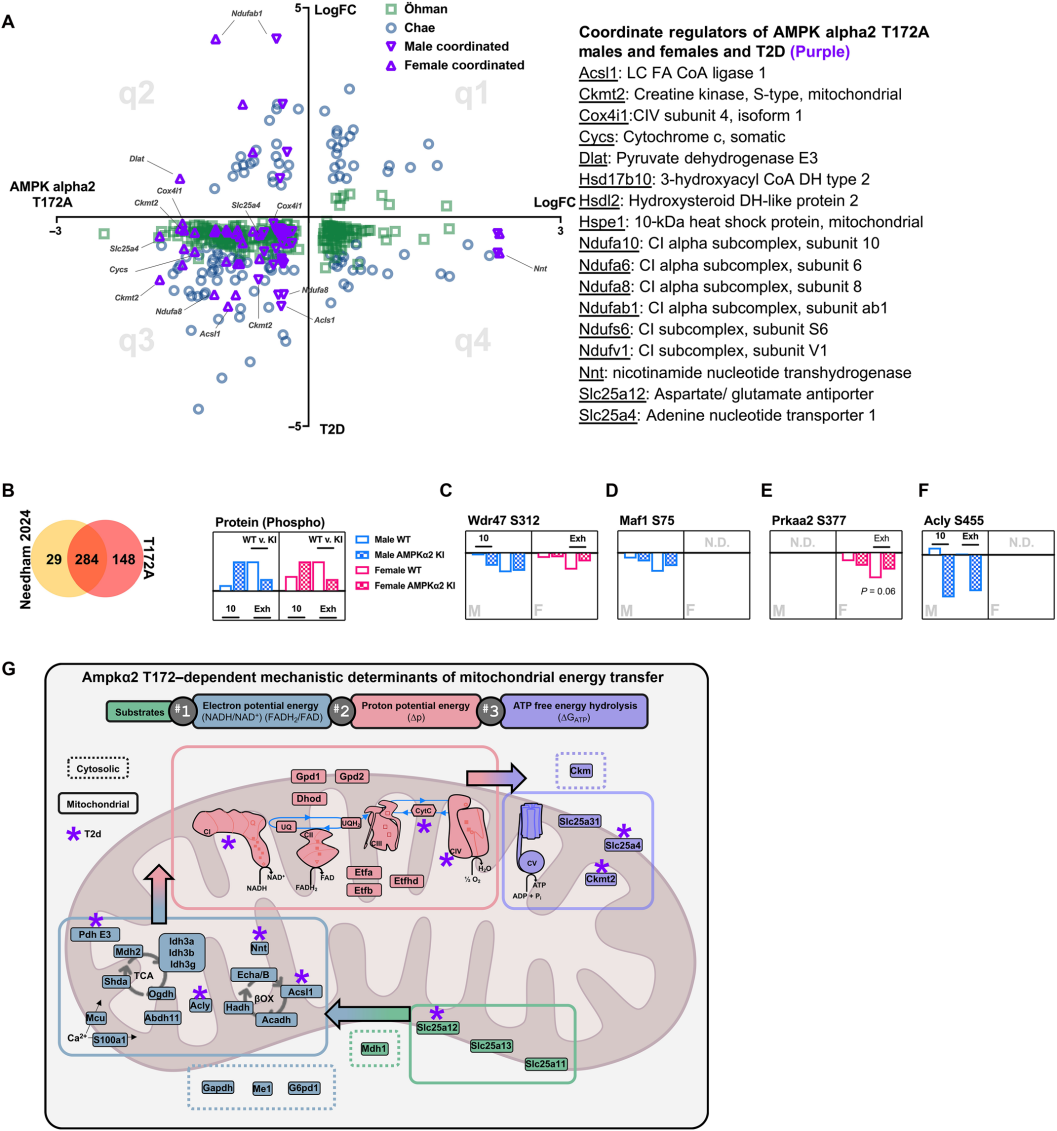
Ampkα2 KI mice share coordinate regulation with type 2 diabetic patient skeletal muscle. (**A**) Global proteomic results for males and females Ampkα2 mice were compared to two datasets of diabetic human skeletal muscle and plotted in four quadrants (q1 to q4) with increased or decreased expression with *x* axis representing Ampkα2 T712A KI results and *y* axis representing the two human studies (Öhman green; Chae blue O). Coordinated Ampkα2 KI study results in the Öhman and Chae datasets were presented as male and female data in purple triangles regardless of quadrant agreement and are listed in the table (right) (total 17 proteins with adjusted *P* < 0.05). (**B**) Overlap between Needham 2024 (*56*) (yellow) and our phosphoproteomic dataset (red). (**C** to **F**) Log_2_FC of phosphorylation at ATP-citrate synthase (Acly) S455, WD repeat domain 47 (Wdr47) S312, Ampkα2 (Prkaa2) S377, and Maf1 S75 in males and females at 10 min (10) and Exh (*P* < 0.05). N.D. not detected. (**G**) Mechanisms of mitochondrial energy transfer separated by regulation of substrate (green), electron potential energy (blue), proton potential energy (pink), and free energy of ATP hydrolysis (lilac). All components in the figure were significantly down-regulated in global proteomic analysis with adjusted *P* < 0.05. Purple asterisk indicates observed presence in T2D datasets from (A) and (B).

We first plotted the coordinated overlap of our global proteomics results with two proteomic datasets from skeletal muscles of patients with diabetes (fig. S7 and Fig. 5A) (*54*, *55*). Proteins that share decreased expression between patients with diabetes and Ampkα2 KI mice (q3) may unveil the putative role of reduced Ampkα2 T172 in T2D. We observed a compelling correlation between patients with T2D and Ampkα2 KI mice that elucidates a reduced expression in the regulation of mitochondrial energy transfer: substrate utilization, transport, and electron transmission [e.g., Slc25a11 (malate aspartate shuttle) and electron transfer flavoproteins Etfa/Etfb)], electron potential energy generation through NADH and FADH_2_ [e.g., Acyl–coenzyme A dehydrogenase medium (Acadm), isocitrate dehyrogenase (Idh3a)], ETC complexes [e.g., Ndufv1 (C1), Sdha (CII), Uqcr10 (CIII), and Cox7a2 (CIV)], and maintenance of phosphorylation potential [e.g., adenine nucleotide translocase (Ant1), phosphate carrier (Slc25a3), and mitochondrial creatine kinase (Ckmt2)] (Fig. 5A). Further, we identified 17 proteins that were present in male and female Ampkα2 KI as well as both T2D studies: Acls1, Ckmt2, Cox4i1, Cycs, Dlat, Hsd17b10, Hsdl2, Hspe1, Ndufa10, Ndufa6, Ndufa8, Ndufab1, Ndufs6, Ndufv1, Nnt, Slc25a12, and Slc25a4 (annotated in purple and listed in table).

Second, phosphoproteomic analysis of insulin-sensitive and insulin-resistant patients with exercise demonstrated significant regulation of 313 unique proteins associated with glucose uptake (*56*), overlapping with 284 of the significantly regulated unique genes from our results (data file S2 and Fig. 5B). However, this comparison demonstrated only four significantly regulated phosphorylation sites between ours and the published study: ATP-citrate synthase (Acly) S455, WD repeat domain 47 (Wdr47) S312, Ampkα2 (Prkaa2) S377, and Maf1 S75 (Fig. 5, C to F). Wdr47 S312, Prkaa2 S377, and Maf1 S75 were significantly altered by exercise at EXH, seemingly independent of Ampkα2 T172 regulation. Contrastingly, Acly S455 was dramatically impaired in male KI mice at 10 min of exercise and EXH, highlighting the potential importance of this site to Ampkα2 T172 signaling and the development of T2D. Collectively, these results demonstrate a significant role of Ampkα2 T172 activation in mediating mitochondrial energy transduction, reduction of which putatively leads to metabolic dysregulation in diabetic skeletal muscle, providing strong rationale for targeting Ampkα2 T172 activation to mitigate diabetic pathology (Fig. 5G; purple asterisk).

## DISCUSSION

Skeletal muscle requires the maintenance of positive energy charge [ATP:ADP] and phosphorylation potential (∆*G*_p_) for its roles in daily function and is highly responsive to physiological changes of bioenergetic demand and proton motive force (∆p) that occur during exercise. It is therefore conceivable that muscle has a sensitive mechanism to detect and react to fluctuation in energetic state, with the purpose of initiating activation of signaling pathways and transcriptional mechanisms that refine bioenergetic form and function. This study reveals that skeletal muscle relies heavily on the energetic bellwether molecule Ampkα2 via its activation by phosphorylation at T172. More than 100 direct Ampk targets have been identified in the literature (*41*, *42*, *57*)—an ever-increasing number—and the current dataset uncovers a litany of novel and vital factors to skeletal muscle metabolism, mitochondrial quantity and quality, and sarcomeric integrity/degradation pathways, among others. Predominantly, our results demonstrate that in the absence of Ampkα2 T172 signaling, skeletal muscle have diminished metabolic flexibility consequently restricting exercise capacity. Herein, we have mechanistically unveiled key regulatory nodes of Ampk in skeletal muscle at baseline and in response to exercise.

A primary objective of the current report is to improve the mechanistic understanding of the role of Ampkα2 signaling, specifically the role of T172 phoshorylation, in skeletal muscle metabolic regulation during exercise. Pursuant to this goal, we compiled data from 25 studies that used genetic means to manipulate Ampkα2 (with or without Ampkα1) (Table 2). Largely, these studies substantiate our results of the negative impact of Ampkα2 ablation on exercise capacity, glucose homeostasis, mitochondrial function, and general metabolism. Of particular interest, previous RNA sequencing analyses similarly implicate transcriptional control of metabolism, mitochondria, and muscle contractile proteins as under regulation by Ampk (*12*). The CRISPR approach is not without limitations of efficiency, delivery, and efficacy (*58*), yet the literature is largely in agreement that Ampkα2 T172 is the primary mechanism of energy sensing within skeletal muscle and is activated by exercise (*27*, *49*, *59*), providing an ideal mechanism for the context of our study.

**Table 2.** Genetic models of AMPK α2 disruption in mice effects in skeletal muscle. References grouped to similar genetic approach. HSA, human skeletal muscle actin; VWR, voluntary wheel running; *J*O_2_, oxygen flux of mitochondrial respiration; TA, tibialis anterior; mt, mitochondrial; SOL, soleus; EDL, extensor digitorum longus; GA, gastrocnemius; QUAD, quadriceps muscle; KD, kinase dead; Tg, transgenic; CI-CIV, ETC complexes; EM, electron microscopy; DG, deoxyglucose.

Reference	Genetic approach	Exercise performance	Glucose regulation	Mitochondrial fitness	Metabolism	Muscle force/properties
(*5*, *9*–*13*)	AMPK α1/2 KO on HSA background, muscle specific	↓ Capacity (VWR) ↓ RER during exercise ↓ fat use ↓ VO_2_ Max ↓ Performance/run to fatigue	↑ GLUT4 expression (QUAD) ↓ Glycogen [c] w/ exercise (QUAD, TA) ↓ TBC1D1 pS237 expression (QUAD) = blood glucose = glucose uptake or ↓ glucose uptake w/ exercise/contraction	↓ Cytochrome C expression = AMP Deaminase activity/sensitivity = *J*O_2_ (TA) or ↓ *J*O_2_ ↑ Oxidative stress, ↓ ETC expression ↓ Citrate synthase expression ↓ ATP [c] = mtDNA	↓ Fatty acid transport ↓ muscle glycogen	= Fiber type (SOL) ↓ Force (SOL/EDL/TA) ↑ Fatigue (SOL)
(*14*)	AMPK α1/2 KO ACTA-Er2-Cre (imdKO)		= Glucose disposal = Glucose uptake w/ contraction (EDL/SOL)			
(*15*–*24*)	AMPKα2 Lysine 45 → Arginine, creatine kinase promotor	↓ Capacity (VWR), ↓ Run to fatigue = VO_2_ or ↓ VO_2_ = RER	= or ↓ glucose uptake w/ exercise, AICAR, or contraction = GLUT4 or ↑ GLUT4 expression baseline or w/ exercise ↓ TBC1D1 expression = pAkt T308 S473 expression	= ATP, lactate, PCr:Cr, AMP:ADP, glycogen [c] at rest and exercise ↓ CI and CIV activity = CI + III, II, II + III activity = citrate synthase activity	↓ pACC S227, S79, S212, S221 expression ↓ muscle glycogen w/ exercise (QUAD) = Body composition = Palmitate oxidation = FA [c] w/ contraction = CPT1/CD36 expression	↓ Force (EDL) ↑ Fatigue (EDL) = Muscle weights (SOL/EDL) = hindlimb fatigue
(*25*–*27*)	AMPKα2 Tg, Aspartate 157 → Alanine, creatine kinase promotor	= Capacity (VWR)	= Glucose transport w/ contraction ↓ AICAR tolerance test = blood glucose	= Creatine phosphate, ATP, ADP, AMP [c] (GA) = PGC1α expression = mtDNA = mt Density (EM) = CytC Expression	= Activity in metabolic cages = pACC S79, rest/exercise	↓ Force production
(*28*–*32*)	AMPKα2 KD Tg: amino acids 189 to 260		↑ Plasma fed glycemia (mg/dl) ↓ plasma fed insulin (ng/dl) ↓ GTT/ITT ↓ 2-DG uptake w/ AICAR = GLUT4 expression (WG/RG) ↓ Hexokinase II expression (WG) = GLUT4 mRNA w/ exercise	↓ Cytochrome C expression ↓ ATP [c] w/ exercise + recovery ↑ AMP [c] 90 min exercise ↓ = PGC1α mRNA exercise + recovery ↓ PDH-E1α site 1/2 expression ↓ ATP [c] w/ exercise ↑ AMP: ADP	= % Fat mass ↓ pACC S227 expression (EDL/SOL) ↓ muscle glycogen	

We first observed that Ampkα2 KI mice activate glycolysis more rapidly during exercise through several lines of evidence: (i) reduced crossover time and earlier increase of RER during VO_2_max testing, (ii) increased glycolytic flux with exercise through rapid utilization of G1P and accumulation of pyruvate, and (iii) greater changes of glycolytic mechanisms observed in phosphoproteomics (e.g., Tbc1d1, Eno3, and Pkn2) during exercise. The disconnect we observed between anerobic and aerobic metabolism appear through Pdh. Several studies of the role Ampk in exercise indicate a regulatory role of Pdh, with some showing an associated increased expression (*60*) and some decreased activity (*32*). Our data elucidate that Pdh (Pdha1, Pdhb, and Pdhx) mitochondrial pyruvate transport proteins (Mpc1/2), as well as Pdh E2 (Dlat) and E3 (Dld) were all diminished in Ampkα2 KI mice, limiting the incorporation of pyruvate into the TCA cycle.

The flow of energy from electron-rich substrates derived from glycolysis to ATP free energy (∆*G*_ATP_) occurs by mitochondrial energy transduction, regulated by three nodes (*47*): (i) matrix dehydrogenases that generate electron potential energy in the form of NADH and FADH_2_, (ii) the electron transport system maintenance of proton potential energy, and (iii) ATP synthesis to maintain ∆*G*_ATP_. In this regard, impaired metabolic and mitochondrial energy transduction is strongly indicated by the proteomic and metabolomic changes we observed in Ampkα2 KI mice related to each of these three nodes (depicted in Fig. 5G): (i) decreased expression of matrix dehydrogenases for cytosolic electron transfer in the form of [NAD^+^], Hadh; (ii) decreased expression of electron transport chain complexes CI, CII, CIII, and CIV for mitochondrial respiration; and (iii) decreased expression of Ckmt2, Ant4, and CV directly involved in ATP synthesis and maintenance. Although Ampkα2 KI mice showed dysfunction at each of these steps, our most comprehensive and mechanistic findings point mainly to the importance of nodes 1 and 2. Further studies are required to dissect discrete mechanisms, yet together, our results present compelling evidence for the regulatory role of Ampkα2 T172 activation in governing bioenergetic function and response in skeletal muscle, which dictates exercise capacity.

The primary findings in this study indicates a robust regulatory control of Ampkα2 T172 in mitochondrial quality and function, which has been highlighted in the literature (*53*) but infrequently mechanistically ascertained. Our results clearly demonstrate that in the absence of Ampkα2 T172 signaling, mitochondria in skeletal muscle are limited in their capacity to import and incorporate nuclear-encoded proteins into the mitochondria reticulum, construct ETC complexes/supercomplexes, and regulate mitochondrial integrity (Fig. 2D and fig. S4, A to D). These defects may collectively limit metabolic capacity. However, what is unclear from the current analysis is whether the decrements to mitochondrial integrity and electron transport chain assembly proteins are causative to or collateral consequences of diminished aerobic metabolism, as the demand of OXPHOS may enable and expediate formation of machinery that facilitates its function. In this regard, there is some evidence that metabolic demand coordinates the assembly and maturation of individual electron transport chain complexes and larger supercomplexes (*61*). Further, Ampk activation through AICAR (5-Aminoimidazole-4-carboxamide ribonucleoside) has recently been used to elucidate the mechanisms integrating ETC assembly and efficiency with metabolic cues (*62*, *63*), for which the current dataset provides the most comprehensive profiling to date. This specific mechanism may be further informed by a recent study from our group (*64*) that demonstrate a novel pool of mitochondria-localized Ampk in skeletal muscle that is responsive to energetic stress.

Our dataset has significant translatable potential as several recent studies have demonstrated that insulin-resistant skeletal muscle has a proteomic signature of decreased OXPHOS machinery, ETC component expression, muscle integrity, and increased proteasomal proteins (*65*, *66*), closely reflected in the expression of these proteins in our Ampkα2 KI mice. Acly S455 phosphorylation was significantly decreased in Ampkα2 KI mice, indicating the importance of this site as downstream of Ampkα2 T172 signaling and vital to metabolism. Further, the 17 key proteins identified have substantial regulatory control over the three nodes of mitochondrial energy transfer: (i) Nnt, Acls1, Slc25a12, and Dlat; (ii) CI and CIV proteins and cytochrome c; and (iii) mitochondrial creatine kinase (Ckmt2) and Slc25a4 (adenine nucleotide transporter 1), underscoring this mechanism as central to diabetic pathology (Fig. 5). Although the global proteomic results for Ampkα2 KI highlight significant regulation at nodes 1 and 2, the overlap with T2D muscle points toward the importance of node 3. In this regard, the activation of Ampk with exercise may serve to mitigate diabetic regulation of the mitochondria, as analysis has shown that exercise training activates mitochondrial signaling pathways that directly oppose the effects of diabetes (*67*).

In conclusion, our results demonstrate that Ampkα2 T172 activation is critical for both glycolytic and oxidative metabolism and exercise capacity in skeletal muscle. Ampkα2 T172 activation is not only required for the maintenance of metabolic machinery but also for the metabolic flexibility during exercise. Specifically, Ampkα2 activation via T172 phosphorylation dictate the expression of each ETC complex, assembly factors, and components of matrix integrity that are crucial for the maintenance of mitochondrial quality and function. Thus, we have provided foundational experimental evidence with intact protein stoichiometry that Ampkα2 activation is intimately involved in regulating of metabolic energy transfer and oxidative phosphorylation at baseline and during exercise. This is a study that comprehensively integrates genetic, phenotypic, and multiomic approaches to provide a high-resolution depiction of the multifaceted roles of Ampkα2 T172 activation in skeletal muscle metabolic and contractile functions. Last, these results position Ampkα2 T172 as a vital regulator that could be augmented in skeletal muscle as a therapeutic targeted against T2D and potentially other diseases.

## MATERIALS AND METHODS

### KI models and animal care

To ascertain the functional role of the Ampk α1 T172 and α2 T172, mutants were developed at the University of Virginia Genetically Engineered Murine Model Core using CRISPR-Cas9 gene editing to replace the T172A to generate a nonactivatable subunit that could not be phosphorylated by upstream kinases. Successful base-pair substitution was confirmed by sequencing and Western blot, and maintenance of these mouse lines were performed by in-house breeding and as previously demonstrated (*64*). For generation of Ampk α1(T172A) KI mice, we used the genomic sequence of the Prkaa1 gene to search (http://crispor.tefor.net/) for the closest PAM site to T172 (T183 in the protein of the mouse gene) and designed forward primer 5′-GAAATTAATACGACTCACTATAGGAATTTTAAGAACAAGCTGGTTTTAGAGCTAGAAATAGCAAG-3′ and reverse primer 5′-AAAAGCACCGACTCGGTGCCACTTTTTCAAGTTGATAACGGACTAGCCTTATTTTAACTTGCTATTTCTAGCTCTAAAAC-3′ for polymerase chain reaction (PCR) and in vitro transcription to obtain single guide RNA (sgRNA). For generation of Ampk α2(T172A) KI mice, we used the genomic sequence of the Prkaa2 gene to search for the closest PAM site and designed forward primer 5′-GAAATTAATACGACTCACTATAGGAATTTCTACGAACTAGCTGGTTTTAGAGCTAGAAATAGCAAG-3′ and reverse primer (the same as above) for PCR and in vitro transcription to obtain sgRNA. We ordered polyacrylamide gel electrophoresis (PAGE)–purified oligo grade repair templates with the change of T172 codon from ACA to GCT for Ampk α1 and ACT to GCT for Ampk α2 from Integrated DNA Technologies (Coralville, IA). The repair template, sgRNA and Cas9 protein were submitted to the core facility for generation of KI mice.

Ampk α1 and α2 T172A KI mice (C57BL/6 background; 8- to 12-week-old) and WT littermate controls were housed in temperature-controlled (21°C) rooms with 12:12 light-dark cycles and were provided standard chow and water ad libitum. Male and female mice were used for each outcome described below with number of animals disclosed in each figure legend and are defined by a circle symbol for females and square for males, where appropriate, to facilitate transparency and abide by SAGER guidelines (*68*). All experimental procedures were approved by the University of Virginia (#3762) and Fralin Biomedical Research Institute (22-251) Institutional Animal Care and Use Committees.

### Body composition measurements

EchoMRI (Houston, TX) was used for measurements of body composition. Following daily calibration with canola oil system test sample, the animals were weighed and scanned individually for body composition including a water stage. Outcome measures included fat tissue (g) and lean mass tissue (g). Weights were normalized to bodyweight for fat content or tibia length collected at end point for lean mass.

### Metabolic cage measurements

Oxymax Comprehensive Lab Animal Monitoring Systems cages (Columbus Instruments, Columbus, OH) were used for indirect calorimetry measures. Following acclimatization, an initial set of animals were individually housed for a 24-hour monitoring period followed by 24 hours of monitoring for outcome measures as analyzed by the Oxymax software and included substrate utilization (VCO_2_, VO_2_, and RER) and assessment of locomotor activity. Activity is measured by infrared beams at a locomotion axis (*X* and *Y*) and rearing axis (*Z*). Total activity indicates the sum of individual beam breaks during an interval, even at the same coordinates, and can be increased by repetitive beam breaks through activities such as grooming. Ambulatory activity measures the breaking of beams at different coordinates and does not measure repetitive breaking of beams at the same coordinates during an interval.

### Metabolic tolerance tests

Metabolic testing included glucose (GTT) and insulin (ITT) tolerance testing with 48 hours in between GTT and ITT. Animals were habituated to handling for 3 days before beginning the tolerance testing to limit glucose release due to distress. Glucose and insulin were prepared with sterile saline and filter sterilized for 2 mg/g and 1 U/kg injections, respectively. The morning of tests (0900), the animals were weighed and individually housed to begin fasting with ad libitum access to water. Following 6 hours (0900 to 1500) of fasting, blood glucose was measured at baseline (0 hours) using a glucometer (milligrams per deciliter; Countour blood glucose meter, Bayer, Leverkusen, Germany) followed by glucose or insulin injection intraperitoneally. GTT and ITT performed subsequent blood glucose measures at 30, 60, and 90 min and 15, 30, 60 min, respectively. Following the final measurement, the animals were returned to their normal housing cages and provided access to food and water and monitored during recovery.

### Measurements of exercise performance and exhaustive exercise procedure

Two separate exercise tests were performed to measure performance via VO_2_max and exercise tolerance (protocols provided in Fig. 1 and at https://fbri.vtc.vt.edu/research/labs/yan/protocols.html).

Exercise tolerance or “run to fatigue” protocol was performed on the Columbus Instruments 3/6 treadmill as previously described (*69*). Treadmill habituation was identical to the VO_2_Max testing. Stages of the exercise endurance testing are depicted in Fig. 1. Mice were tested for blood lactate (millimole per liter) before beginning (L_0_) with follow up testing after completing the test (L_End_) to confirm EXH. Endurance testing begins with 13.41 m/min treadmill speed for 0 to 30 min and increasing to 16.09 m/min (30 to 60 min), 18.78 m/min (60 to 90 min), 21.46 m/min (90 to 120 min), 24.14 m/min (120 to 150 min), and 26.82 m/min (>150 min) and remained at this speed until EXH was confirmed on the shock grid as described above and by L_End_.

VO_2_Max was assessed with a metabolic treadmill (Columbus Instruments) and analyzed via Oxymax software. The animals were acclimated to the treadmill over 3 days immediately before testing and included a 10-min bout at 10 m/min 5° incline including shock. Following daily calibration, at the beginning of the light cycle, the animals were weighed and placed into individual running compartments set to 5° incline. Protocol for VO_2_Max is demonstrated in Fig. 1 with a sampling rate of 3 min. The animals begin at 0 m/min for 2 min (stage 1, white bar), followed by 12 min of 5 m/min “warm up” period (stage 2, gray bar) after which the test begins. Stage 3 consists of 3 min of 10 m/min followed by 4 min of 13 m/min (stage 4, black bar). All subsequent stages (black bar) are 3 min and increase by 3 m/min with each stage. EXH is defined as an inability or unwillingness to continue running as observed by five sequential shocks on the grid at the back of the chamber. When the test is completed, the shock grid is turned off and continuous reading of two additional cycles is recorded and the test completed. The animals were returned to their cages and monitored during recovery.

Exhaustive exercise was performed identically to the exercise tolerance test with similar determination of end point. The first cohort of animals were taken after 10 min during which they were immediately euthanized, and skeletal muscle was collected, frozen in metal clamps, cooled with liquid nitrogen, and then stored at −80°C until time of multiomic analysis. A second cohort of animals was euthanized in a similar fashion following determination of EXH. Blood lactate was collected as described above to confirm EXH.

### Tissue preparation and Western blot

Mice were euthanized under 2.5% isoflurane at the time of skeletal muscle harvest following an overnight fast. The plantaris, gastrocnemius, soleus, and heart tissues were removed with surgical tools rinsed in 1× phosphate-buffered saline, weighed, and prepared for different assays accordingly. For Western blot, tissues were homogenized in glass homogenizers with protein sample buffer containing 50 mM tris-HCL (pH 7.4), 0.01% bromophenol blue, 10% glycerol, 1% SDS, 127 mM 2-mercaptoethanol, and 20 mM dithiothreitol (DTT), supplemented with protease inhibitor cocktails and phosphatase inhibitor cocktail tablets (Sigma-Aldrich). Homogenized tissue lysates were then boiled in a heat block at 98°C for 5 min and stored in −70°C. Protein concentration was determined by the RCDC protein assay kit (Bio-Rad).

Protein lysates were subjected to SDS-PAGE at 100 V for 1 hour and transferred onto nitrocellulose membranes at 80 V for 2 hours. Membranes were probed with the following primary antibodies at a 1:1000 dilution: targeting phospho-Ampkα (T172) (CST, #2535), Ampk α (CST, #2532), phospho-ACC (CST, #3661), phospho-ULK1(CST, #5869), ULK1 (Sigma-Aldrich, #A7481), α-tubulin (Abcam, Cambridge, MA, USA, #ab11304), glyceraldehyde-3-phosphate dehydrogenase (CST, #2118) and normalized to a muscle common standard loaded in the middle of the membrane. Secondary antibodies were goat anti-rabbit IR800 and anti-mouse IR680. Membranes were scanned using the odyssey infrared imaging system (LICOR). Proteins were analyzed and normalized to a common protein standard loaded on gel.

### Collection of mitochondria enriched fraction

Plantaris muscle was separated from the soleus and gastrocnemius, weighed, and immediately placed in 1 ml of ice-cold MIB [bovine serum albumin (BSA, 2 mg/ml), sucrose (70 mM), mannitol (210 mM), Hepes (5 mM), and EGTA (1 mM, pH 7.1)]. At 4°C, muscle was processed with a saw-tooth homogenizer for ~30 s and spun at 4°C for 10 min at 800*g* to pellet cellular debris. Supernatant was collected and spun again at 9000*g* at 4°C for 10 min. Supernatant following this spin was collected as cytosolic fraction, and the remaining fraction was resuspended in MIB without BSA for quantification of mitochondrial protein by the Bradford method. Protein loading for respirometry was normalized to mg of mitochondrial protein.

### Mitochondrial respiration

High-resolution respirometry was used to assess mitochondrial oxygen consumption in an Oroboros O2k (Innsbruck, Austria). We used the creatine kinase (CK) clamp method during this assay. CK functions to maintain stoichiometric ratio of ATP:ADP during respiration (creatine + ATP phosphocreatine + ADP), which increases the physiological relevance and interpretation of mitochondrial function in contrast to traditional assays that use a damaging bolus of ADP. This method provides a discrete and comprehensive measure of mitochondrial oxygen consumption predicated on the regulation of energy demand and energy charge as described previously (*47*, *70*) and depicted in fig. S4.

O2k settings remained at 37°C and 500 rpm in 0.5-ml chambers to daily calibration with buffer D {KMES [(2-(N-Morpholino)ethanesulfonic acid potassium salt, 4-Morpholineethanesulfonic acid potassium salt] (105 mM), KCl (30 mM), EGTA (1 mM), KH_2_PO_4_ (10 mM), MgCl_2_-6H2O (5 mM), and 0.05% BSA (pH7.1), solubility factor 0.966} to assess R1 (air saturation, ~200 μM O_2_) and closed chamber R0 (zero oxygen) with sodium hydrosulfide (Sigma-Aldrich, S1256). Chambers were then washed with DI water (5 min for five cycles) before beginning experiments. Oxygen flux (*J*O_2_; pmol/s) was assessed in buffer D supplemented with creatine monohydrate (5 mM) and 20 mg of mitochondrial protein. State 2 respiration (leak state) was assessed with pyruvate (5 mM) and malate (2.5 mM) followed by maximal respiration (∆*G*_ATP_ −12.94 kCal/mol) with CK (20 mM), phosphocreatine (1 mM), and ATP (5 mM). Cytochrome c (0.005 mM) was used to assess mitochondrial integrity with a threshold of 15% increase in respiration. Sequential PCr titrations were then added at 1 mM (∆*G*_ATP_ −13.38 kCal/mol), 2 mM (∆*G*_ATP_ −13.71 kCal/mol), 4 mM (∆*G*_ATP_ −14.12 kCal/mol), and 6 mM (∆*G*_ATP_ −14.45 kCal/mol).

Calculations of ∆*G*_ATP_ were performed using an online calculator [provided in (*47*)] under the conditions of 37°C, 170 mM ionic strength, 5 mM creatine, and 10 mM phosphate (pH 7.1). The plotting of *J*O_2_ versus ∆*G*_ATP_ across the linear force-flow relationship allows for calculation of mitochondria “conductance.” Conductance measures the inverse of resistance (~1/R) of the mitochondrial proton motive force (∆p; voltage) in relationship to Ohm’s law (*I* = V/R) where current (*I*) measures *J*_H+_.

### LC-MS/MS proteomics and phosphoproteomics

Muscle tissue samples from male and female WT and KI mice were collected before exercise (i.e., sedentary control, SED), after 10-min running (10 min), and after EXH and stored at −80°C until processing. Sample processing for global proteomics and phosphoproteomics analysis was conducted as previously described (*71*) with a several modifications. Briefly, frozen muscle tissue samples were minced into small pieces on a prechilled aluminum tray with dry ice. Approximately 25 mg of tissue was collected and homogenized in cold lysis buffer [50 mM tris, 8 M urea, 75 mM NaCl, 1 mM EDTA, aprotinin (2 μg/ml), leupeptin (10 μg/ml), 1 mM phenylmethylsulfonyl fluoride, 10 mM NaF, 1% phosphatase inhibitor cocktail 2, and 1% phosphatase inhibitor cocktail 3 (pH 8.0)] on a prechilled bead beater using 2-min cycle. Samples were then centrifuged at 13,000*g* at 4°C for 10 min to remove debris. Extracted protein was quantified by BCA assay. The same quantity of protein was aliquoted and reduced with 5 mM DTT for 1 Ampk shaking on a thermomixer at 37°C, 1000 rpm. Proteins were then alkylated with 20 mM iodoacetamide with shaking in dark at room temperature, 1000 rpm for 45 min. Samples were diluted fourfold and then digested with trypsin (enzyme to protein ratio = 1:50) for 1 hour, shaking at 1000 rpm, 37°C. A fresh aliquot of trypsin (enzyme to protein ratio = 1:50) was added after the samples were diluted twofold. Then, the digestion was conducted at 37°C 1000 rpm overnight. The digest was desalted by Sep-Pak C18 SPE cartridges (Waters, Milford, MA). Clean peptides were quantified by BCA assay. Two hundred and fifty micrograms of peptides from each sample was aliquoted and concentrated in a speedvac to completely dry to be used for TMT labeling.

Peptides were resuspended in 500 mM Hepes (pH 8.5) at 5 μg/μl. Three replicates of male or female muscle tissue samples from each condition (WT-SED, WT-10 min, WT-EXH, KI-SED, KI-10 min, KI-EXH) were labeled with two TMT18 plexes. TMT reagents were resuspended in anhydrous acetonitrile at 20 μg/μl and added to each sample at a 1:2.5 (peptide:TMT) ratio. Labeling was conducted at 25°C, 850 rpm for 1-hour shaking on a thermomixer. Then, the reaction was quenched by hydroxylamine. Samples from each plex were combined and concentrated in a speedvac, followed by C18 SPE cleanup. The clean TMT-labeled samples were then fractionated into 12 fractions using high-pH reversed phase separation. Five percent of each fraction was used for global proteomics analysis, and the remaining fractions were subjected to immobilized metal affinity chromatography phosphoenrichment using freshly prepared Fe^3+^–nitrilotriacetic acid–agarose beads.

Both global and phosphopeptide fractions were analyzed using Waters nanoAcquity UHPLC system (with 20 cm by 75 μm i.d. 1.9-um column packed in-house with Waters BEH C18 silica) coupled to a Orbitrap Fusion Lumos (Thermo Fisher Scientific, San Jose, CA) with a 120-min LC gradient. Positive ion mode spray voltage was set at 2.2 kV. Full mass spectrometry (MS) spectra were recorded at resolution of 60 K with scan range 350 to 1800 mass/charge ratio (*m*/*z*). Automated gain control (AGC) value was set 4 × 10^5^. Tandem MS (MS/MS) was acquired in data-dependent acquisition mode (DDA) at a resolution of 50 K, AGC of 1 × 10^5^. Isolation window was 0.7 *m*/*z*. High-energy collision dissociation with a normalized collision energy setting of 30% was used. Dynamic exclusion time was set at 45 s.

### Untargeted metabolomics

Muscle tissue samples from male and female WT and KI mice were collected as described above. For extraction, tissues were thawed on ice, and approximately 750 μl of a cold chloroform:methanol (1:2) mixture was added, along with four steel balls (Fisher Brand; diameter, 2.4 mm). Tubes were plunged into liquid nitrogen for 5 min and vigorously shaken in a Fisher Brand Bead Mill 24. Tubes were then vortexed and shaken at 900 rpm for 30 min at 4°C in a temperature-controlled thermal shaker. After adding 400 μl of water, the samples were vortexed, and the upper aqueous phase was recovered as the metabolite mixture. Two hundred microliters of the extract were dried overnight in a SpeedVac and reconstituted in 200 μl of 0.1% formic acid containing the 100X Metabolomics QReSS Kit (Cambridge Isotopes, MSK-QRESS-KIT). A 10-μl aliquot from each tube was removed to create a pooled QC sample, which was injected at the beginning and end of the MS sequence. Additional QC samples were injected after every five sample injections.

Liquid chromatography–MS (LC-MS) data acquisition was performed using a fully automated AcquireX Intelligent Data Acquisition Workflow with additional instrument parameters, and data acquisition and data processing workflow is based on a previous publication (*72*). We first generated an exclusion list generated from a reagent blank sample to determine the background. A pooled QC sample was injected for feature detection and component assembly to create an inclusion list. Using the inclusion list, a series of iterative DDA injections were performed, with each injection informed by the previous one. Precursors from the inclusion list were fragmented, and once detected, they were automatically transferred to the exclusion list. This approach minimized redundant fragmentation and maximized relevant spectra and metabolite annotation. Samples were analyzed on a Thermo Orbitrap IDX Tribrid MS coupled to a Thermo Vanquish UHPLC. Metabolite separation was achieved using a Waters BEH C18 column (Waters Corp.; 2.1 mm by 150 mm, 1.7 μm) maintained at 30°C. Samples were analyzed in both positive and negative ionization mode.

### Differential analysis

Global proteomics and phosphoproteomics were searched against protein sequences from the 2023 UniProt database with the MS-GF+ tool (*73*, *74*). After removal of contaminant proteins, correction for isotope selection error, and phosphosite localization with the Ascore method (*75*), peptide-spectrum matches (PSMs) were filtered to limit the peptide-level false discovery rate (FDR) to 1%. This was followed by a 1% protein-level FDR filter for global proteomics, or the mapping of phosphopeptides to phosphosites and limiting the phosphosite-level FDR to 1%. Decoy PSMs were removed and the parsimonious set of proteins was determined by preferentially assigning peptides to the protein with the highest number of total peptide identifications. For phosphoproteomics, phosphosites were first assigned to any proteins detected in the global proteomics results before assigning them to the protein with the most peptide identifications. The MASIC intensity data were then combined with the processed MS-GF+ search results and intensities were aggregated at the level of proteins or phosphosites and log_2_ transformed (*76*). Since males and females were measured in different TMT plexes, they were separated, and any proteins or phosphosites that were not measured in all samples for a particular sex were removed. Then, samples were normalized by subtracting their median intensities to remove differences due to sample loading and each protein or phosphosite was median-centered.

For the global proteomics, phosphoproteomics, and metabolomics datasets, we fit a linear model that included a single predictor with all combinations of group (KI and WT) and timepoint (SED, 10 min, EXH) as categories using the lmFit function from the limma R/Bioconductor package (*77*–*79*). For global proteomics, the presence of several high variance samples led to the use of sample-specific quality weights; these weights, generated automatically with the arrayWeights function from limma, reduce the contribution of samples with high variance without reducing the available degrees of freedom (*74*). Contrasts were constructed to test for differences between the SED KI and SED WT groups in global proteomics. The phosphoproteomics contrasts compared the 10 min and EXH time points to the SED time point within both KI and WT groups, in addition to testing for differences in the EXH versus SED response between the KI and WT groups: (KI.EXH - KI.SED) - (WT.EXH - WT.SED). Last, the metabolomics contrasts included the four trained versus SED contrasts from the analysis of the phosphoproteomics data and the SED KI versus SED WT comparison that was tested for the global proteomics data. For all omes, the eBayes function from limma was used to squeeze the residual variances toward a global trend that was made robust to the presence of any outlying features (*78*, *80*). *P* values were adjusted across the trained versus SED contrasts and separately for the other contrasts using the method of Benjamini and Hochberg to control the FDR (*81*). *P* values were adjusted across the trained versus SED contrasts and separately for the other contrasts using the method of Benjamini and Hochberg to control the FDR.

### Enrichment analysis

The global proteomics, phosphoproteomics, and metabolomics differential analysis results were each transformed into matrices of *z*-scores (calculated from moderated *t* statistics) separated by sex with gene symbols (proteomics), singly phosphorylated sites (phosphoproteomics), or Reference Set of Metabolite Names (RefMet) metabolite identifiers as rows and contrasts as columns (*82*). In the phosphoproteomics matrices, only the exercise versus SED contrasts were used. To resolve situations where multiple proteins map to the same gene symbol or separation of multiply-phosphorylated phosphosites results in multiple rows for the same site, the feature with the most extreme *z*-score (smallest *P* value) was selected per contrast. If any proteins did not map to a gene symbol, the protein identifier was used to avoid the unnecessary removal of data. This procedure resulted in a total of 3365 genes, 17,636 phosphosites, and 452 RefMet metabolite identifiers from either sex.

Version 2023.2 of the mouse Molecular Signatures Database, particularly the C5 collection of 10,662 GO terms, was selected for the analysis of the global proteomics data (*83*–*86*). Of these terms, 3042 contained at least 10 genes that overlapped with the set of 3365 genes measured in the proteomics data. These ~3000 gene sets were then clustered according to their Jaccard similarity coefficients using the clusterSets function from the TMSig R package to identify groups of highly similar gene sets (*87*). The largest gene set in each cluster was selected as the representative for testing. If two or more gene sets had the same maximum size, the set with the highest proportion of original genes was selected; further ties were broken by selecting the set with the shortest description (often, the most general term) and then the first set alphabetically. This removed 194 redundant gene sets.

The “Kinase_Substrate_Dataset” file was downloaded from PhosphoSitePlus (v6.7.1.1), and the data were filtered to mouse kinases and substrates (*88*). The phosphosites, formed by concatenating the SUB_GENE and SUB_MOD_RSD columns, were then grouped by their known kinases. Only 26 of 239 possible kinase sets contained at least three phosphosites that overlapped with the set of 17,636 phosphosites from the male or female phosphoproteomics datasets.

Metabolite subclasses from the RefMet database were selected for the analysis of the metabolomics data (*82*). Only nine subclasses contained at least 10 metabolites that overlapped with the set of 452 measured metabolites.

Gene sets, kinase sets, and RefMet subclasses were analyzed with the parametric version of the CAMERA-PR test (*45*). This test is a modification of the two-sample *t* test that accounts for correlation between molecules within sets to better control the type I error rate. The analysis was carried out on the *z*-score matrices with the cameraPR.matrix function from TMSig (*87*). As with the differential analysis results, *P* values were adjusted across contrasts using the BH method (*81*).

### AMPK consensus motif mapping

Each peptide was 15 amino acids in length, centering on the phosphorylated residue (position 0). Motif detection was performed on the basis of known AMPK substrate preferences (*52*). Three levels of stringency were applied: strict, relaxed, and minimal motif match. The pattern for strict match was [I/V/L/M] - X - [R/K] - X - X - [S/T] -X - X - X - [I/F/L/M], in which the −5 position must be I, V, L, or M, −3 must be R or K, and +4 must be I, F, L, or M. The pattern for relaxed match was X - X - [R/K] - X - X - [S/T] - X - X - X - [I/F/L/M]. For broader detection, a minimal motif was annotated requiring the presence of a basic residue at −3 with the pattern of X - X - [R/K] - X -X - [S/T] - X - X - X - X - X.

### Statistical analysis (non-omic results)

Data are represented as means ± SEM. In analyses where one variable was present, data were analyzed with a Student’s *t* test. Two-way analysis of variance (ANOVA) testing was performed for several outcomes where multiple variables were present to demonstrate main effects. Statistical significance was established a priori as *P* < 0.05 or *P* < 0.01 where indicated. Data presented as means ± SEM. Statistical analysis performed by *t* test between groups: *P < 0.05, **P < 0.01, ***P < 0.001, and ****P < 0.0001.
