# Supplementary material for: Ampk alpha2 T172 activation dictates exercise performance and energy transduction in skeletal muscle
Source: Sci Adv. 2026 Feb 25;12(9):eaeb3338. doi: 10.1126/sciadv.aeb3338 (PMC12935046; doi:10.1126/sciadv.aeb3338)
Supplement: Supplementary file 1 — Figs. S1 to S7 Legends for data files S1 to S5 [file sciadv.aeb3338_sm.pdf]

Science Advances
AAAS

Supplementary Materials for

Ampk alpha2 T172 activation dictates exercise performance and energy transduction in skeletal muscle

Ryan N. Montalvo *et al.*

Corresponding author: Ryan N. Montalvo, ryanmontalvo@vtc.vt.edu; Zhen Yan, zhenyan1@vtc.vt.edu

Sci. Adv. **12**, eaeb3338 (2026)
DOI: 10.1126/sciadv.aeb3338

The PDF file includes:

Figs. S1 to S7
Legends for data files S1 to S5

Other Supplementary Material for this manuscript includes the following:

Data files S1 to S5

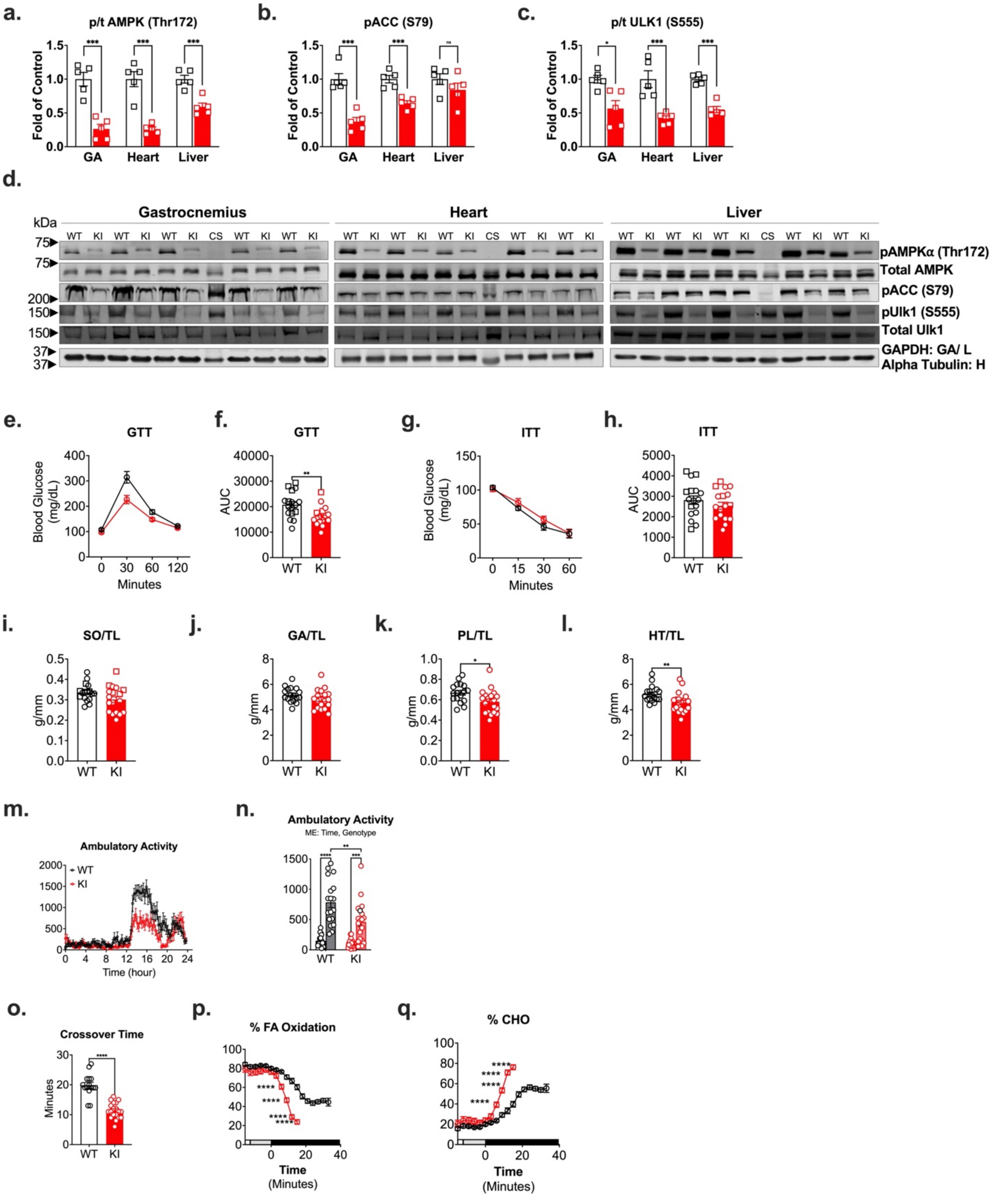

Figure S1: Supplement: Ampkα2 KI mice demonstrate altered metabolic regulation and limited exercise capacity.

(**A-D**) western blot quantification for genotype confirmation (p/t AMPK) and classical Ampk targets (pACC S79; p/t Ulk1-S555) in the gastrocnemius (GA), heart (H), and liver (L). (**E-H**) Glucose (GTT) and insulin tolerance testing (ITT) and area under the curve (AUC) analysis. (**I-L**) Skeletal muscle weights at the time of sacrifice: soleus (Sol), gastrocnemius (GA), plantaris (PL),

and heart (HT) normalized to tibia length (TL; mm). (**M/N**) Metabolic cage measurements (Columbus Instruments) were taken over 24 hours for ambulatory activity. Comparisons made between the light/ resting (0700-1900; time 0-12 hours) and dark/ active (1900-0700; time 12-24 hours) cycles. (**O-Q**) VO_2Max testing evaluation of crossover time to reach anaerobic threshold as demonstrated by percent fatty acid (%FA) oxidation and percent carbohydrate (%CHO) oxidation. Males (n=6 WT; n=7 KI) represented in squares and females (n=11 WT; n=12 KI) in circles for all outcomes; circles represent genotype average for **E/G/P/Q**. Data presented as mean ± SEM. Statistical analysis performed by t-test between groups. Two-way ANOVA performed for **N** for main effects (ME) of time and genotype. Significance indicated as $p < 0.05$ (*), $p < 0.01$ (**), $p < 0.001$ (***), and $p < 0.0001$ (****), ns= not significant.

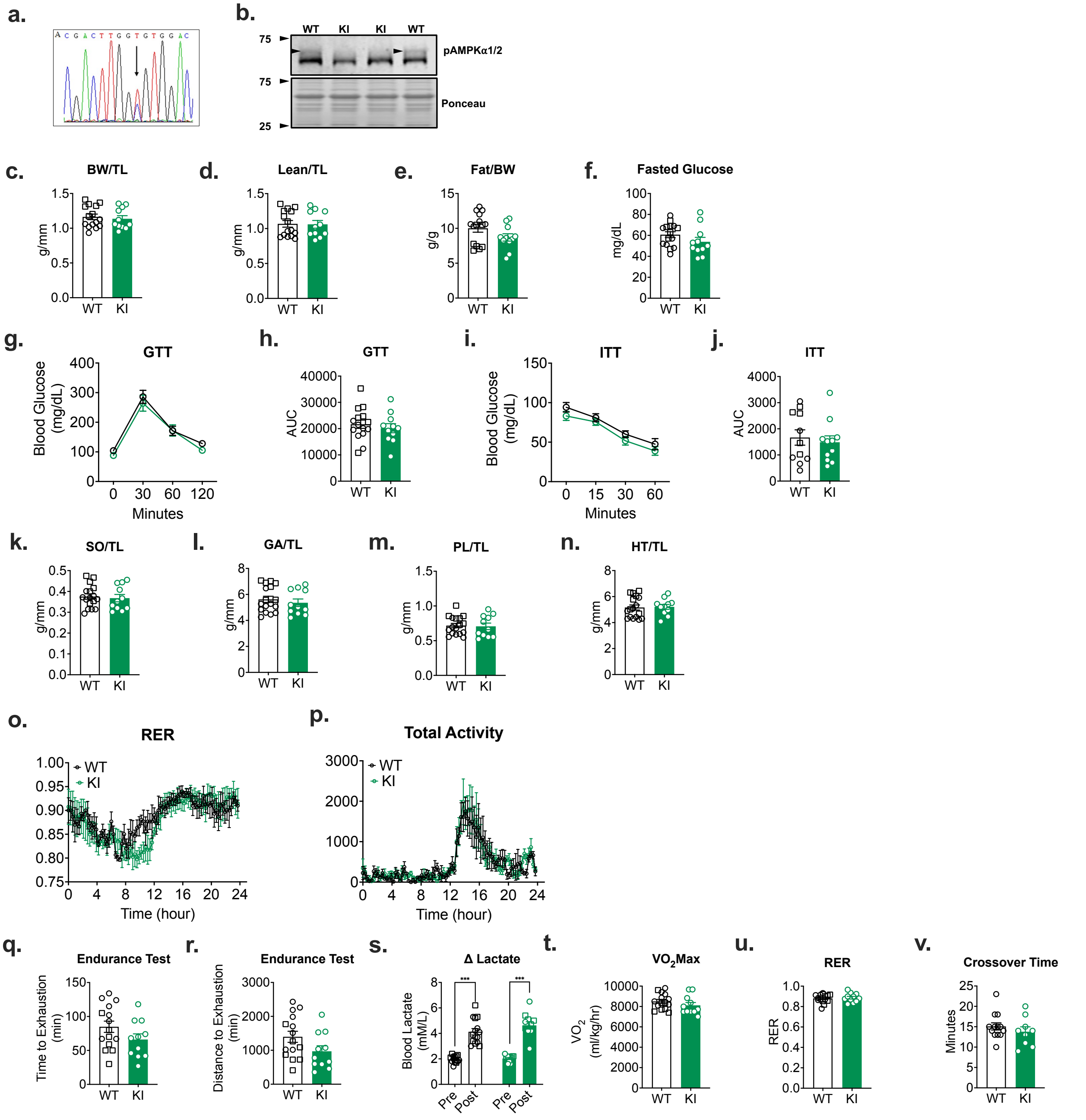

Figure S2: Supplement: Ampkα1 KI mice present normal metabolic and exercise capacity

(**A/B**) Genotype was confirmed with sequencing and western blot for the T>A mutation at Ampkα1 T172 site. (**C-E**) Body composition analysis (EchoMRI) evaluated bodyweight (g) as well as lean mass (g) normalized to tibia length (TL; mm) and fat mass (g) normalized to bodyweight (g). (**F**) Fasted glucose measurements were taken after an overnight fast (1900-0700). (**G-J**) Classical glucose (GTT) and insulin tolerance testing (ITT) and area under the curve (AUC) analysis. (**K-N**) Skeletal muscle weights (g) at the time of sacrifice: soleus (Sol), gastrocnemius (GA), plantaris (PL), and heart (Ht) normalized to tibia length (TL; mm). (**O/P**) Metabolic cage measurements (Columbus Instruments) were taken over 24 hours for total activity and respiratory exchange ratio (RER). Endurance testing and VO_2Max testing performed to evaluate exercise capacity (protocol

indicated in **figure 1**). (**Q-S**) Endurance testing evaluated in minute and meters and confirmed exhaustion by pre-post lactate measurements. VO_2Max testing evaluation of VO_2Max, RER, and crossover time to reach anaerobic threshold. Males (n=4-7 WT; n=4-6 KI) represented in squares and females (n=8 WT; n=5 KI) in circles for all outcomes; circles represent genotype average for **G**/**I**/**O**/**P**. Black/ white indicates WT and green indicates Ampkα1 KI. Data presented as mean ± SEM. Statistical analysis performed by t-test between groups. Significance indicated as $p < 0.05$ (*), $p < 0.01$ (**), $p < 0.001$ (***), and $p < 0.0001$ (****), ns= not significant.

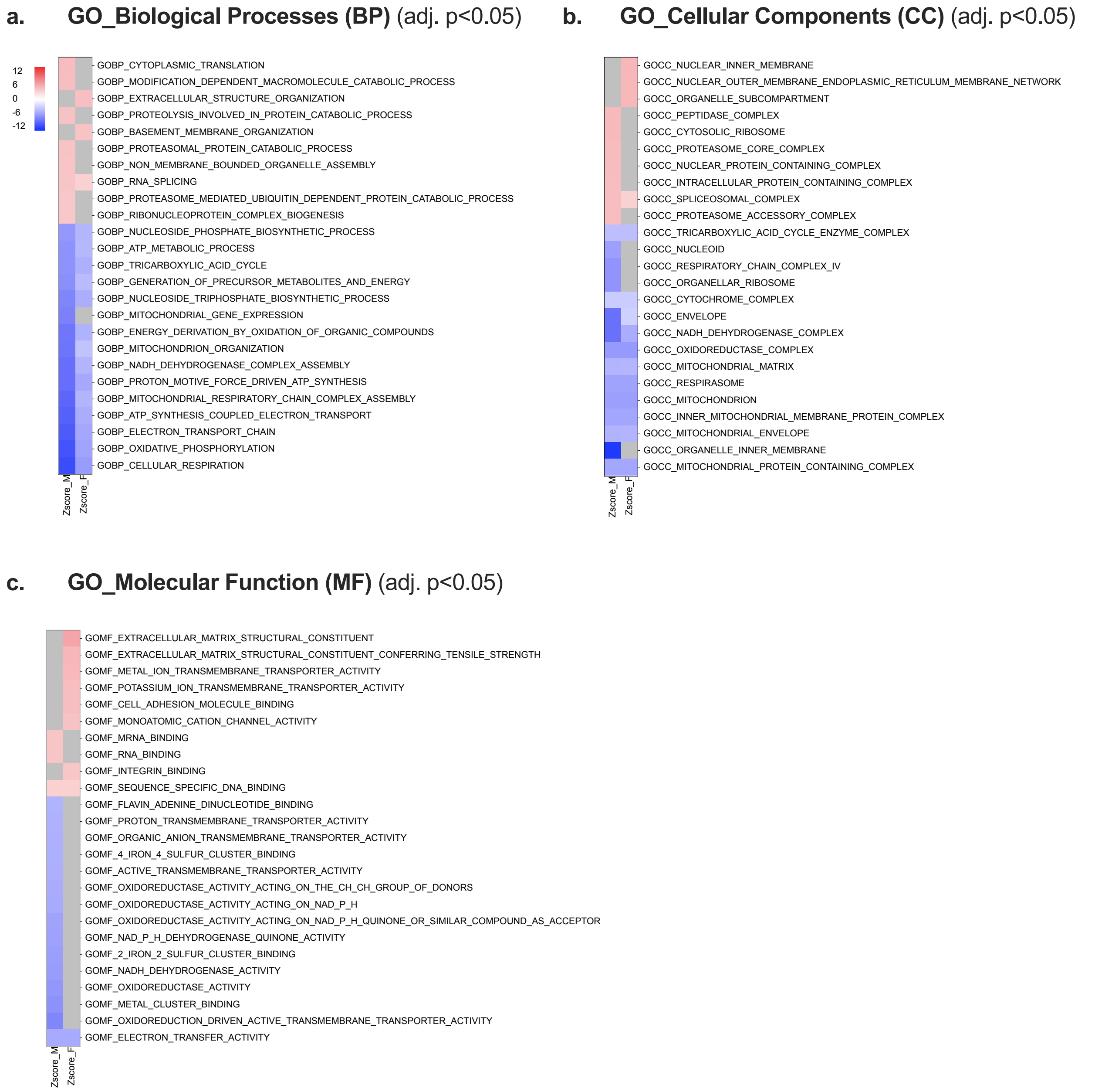

Figure S3: Supplement: Results of global proteomics separated by gene ontology (GO) terms. (**A-C**) Top 10 significantly upregulated (red) and top 15 significantly downregulated (blue) terms for GO terms (GO biological processes (BP); cellular components (CC) and molecular function (MF) represented by Z score. Gray box indicates no result. All results are adjusted $p<0.05$. Full results present in Supplemental data file 1.

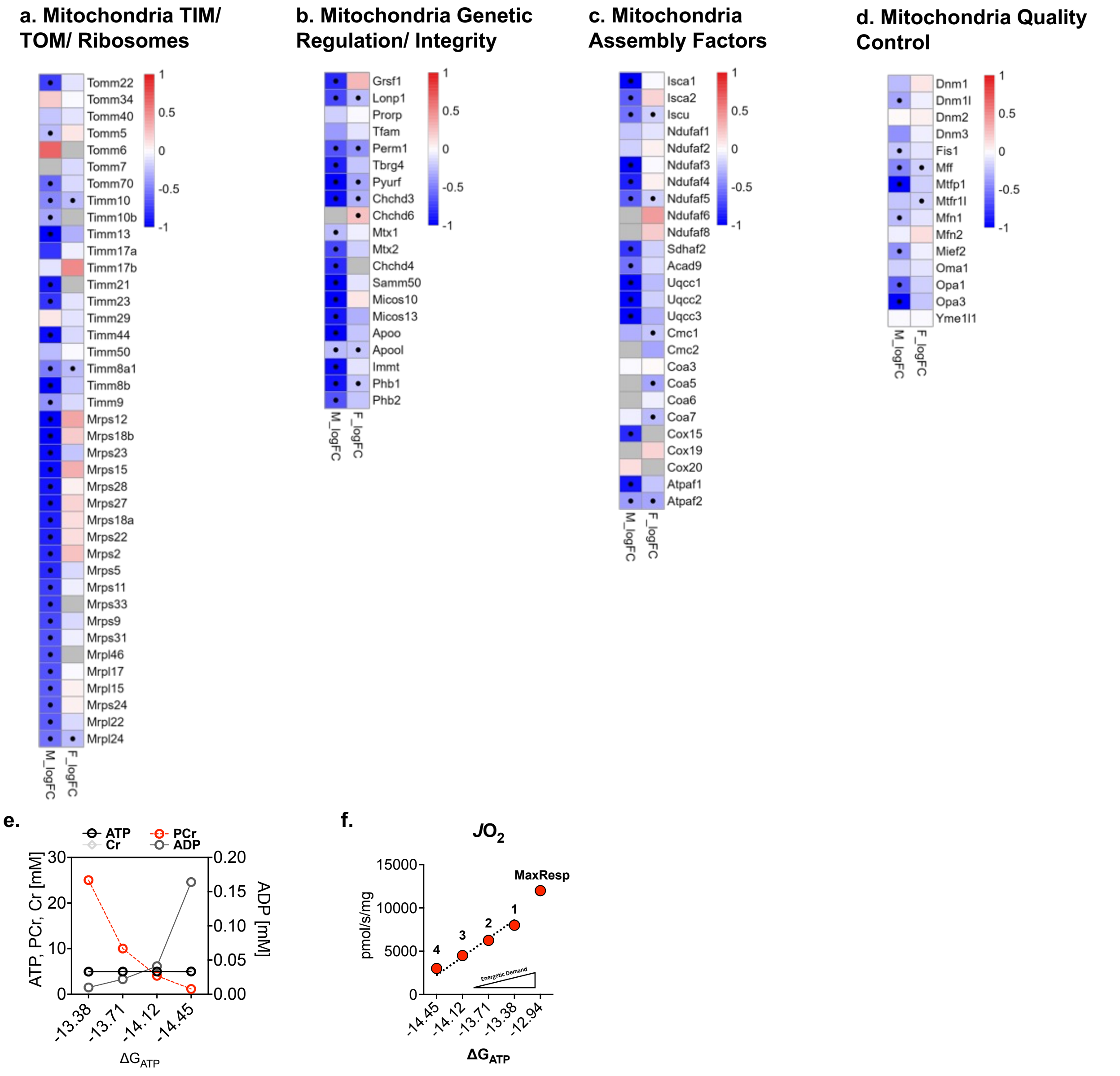

Figure S4: Supplement: Enrichment analysis of global proteomics with MitoCarta overlay, creatine kinase clamp method illustration. Full results present in S**upplemental data file 1**. Red indicates increased expression and blue decreased with • representative of adj. $p < 0.05$ difference within heatmap by t-test (WT n=6; KI n=6). (**A**) Global proteomic processes related to mitochondrial translocase of inner membrane (TIM), translocase of outer membrane (TOM), and representative mitochondrial ribosome proteins (Mrps/Mrpl). (**B**) regulation of genetic processes within the mitochondria and mitochondrial gene transport/processing. (**C**) mitochondria/ electron transport chain assembly factors. (**D**) mitochondrial quality control. (**E/F**) Representative concentrations of ATP, phosphocreatine (PCr), creatine (Cr), and ADP during the creatine kinase

clamp evaluation of respiration and titration of PCr to represent conductance over a range of energy demands. Statistical analysis performed by t-test between groups: adjusted $p < 0.05$ (•).

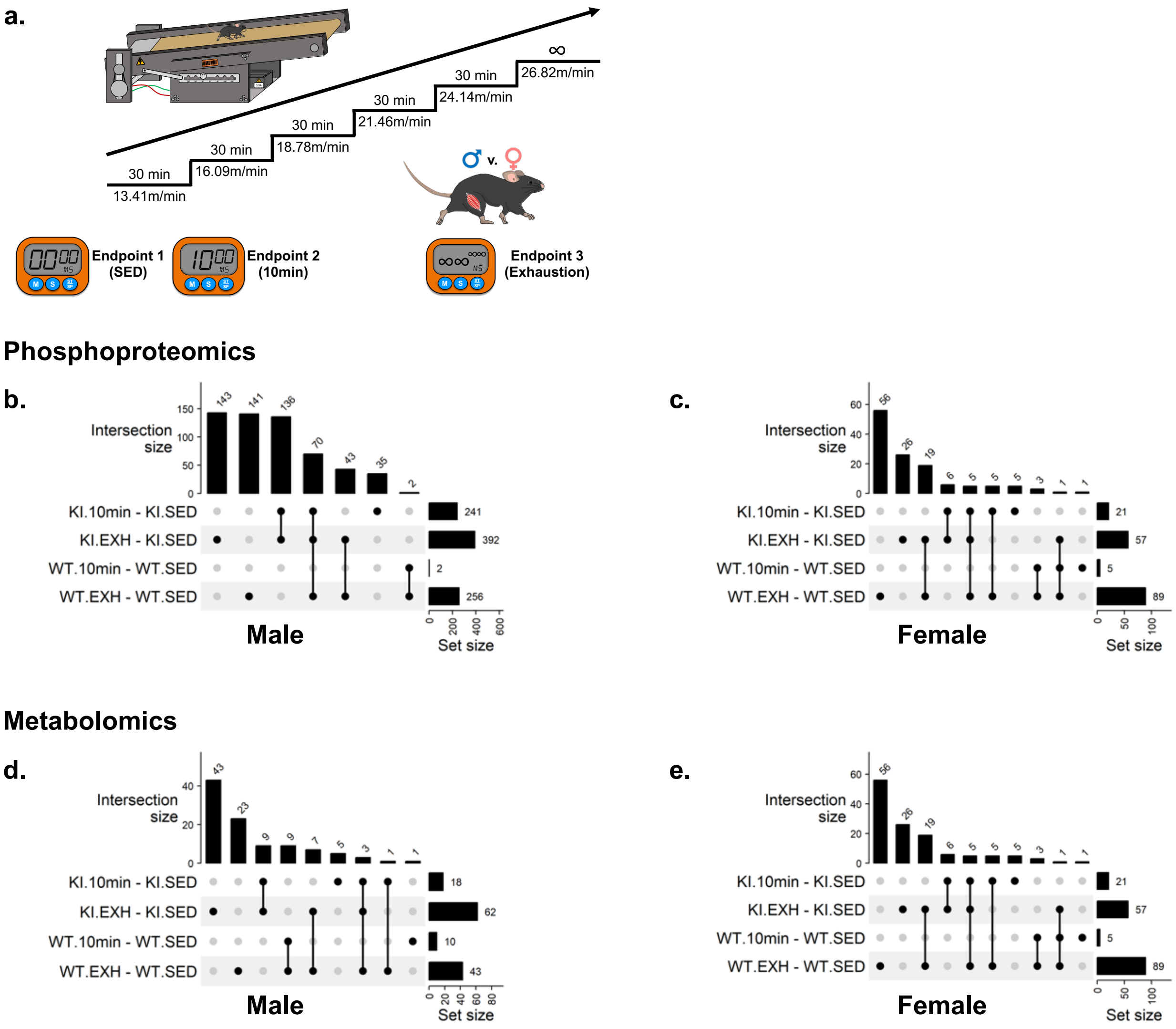

Figure S5: Supplement: Ampkα2 T172 dictates the phosphoproteomic and metabolomics response to exercise within skeletal muscle.

(**A**) representative method of the exhaustive exercise protocol with endpoints at 0 minutes of exercise (SED), 10 minutes of exercise (10) and exhaustion (∞) (WT n=6; KI n=6). (**b/c**) Upset plots of statistically significant male and female phosphoproteome following 10 minutes of exercise and at exhaustion separated by genotype. (**D/E**) Upset plots of statistically significant male and female metabolome following 10 minutes of exercise and at exhaustion separated by genotype.

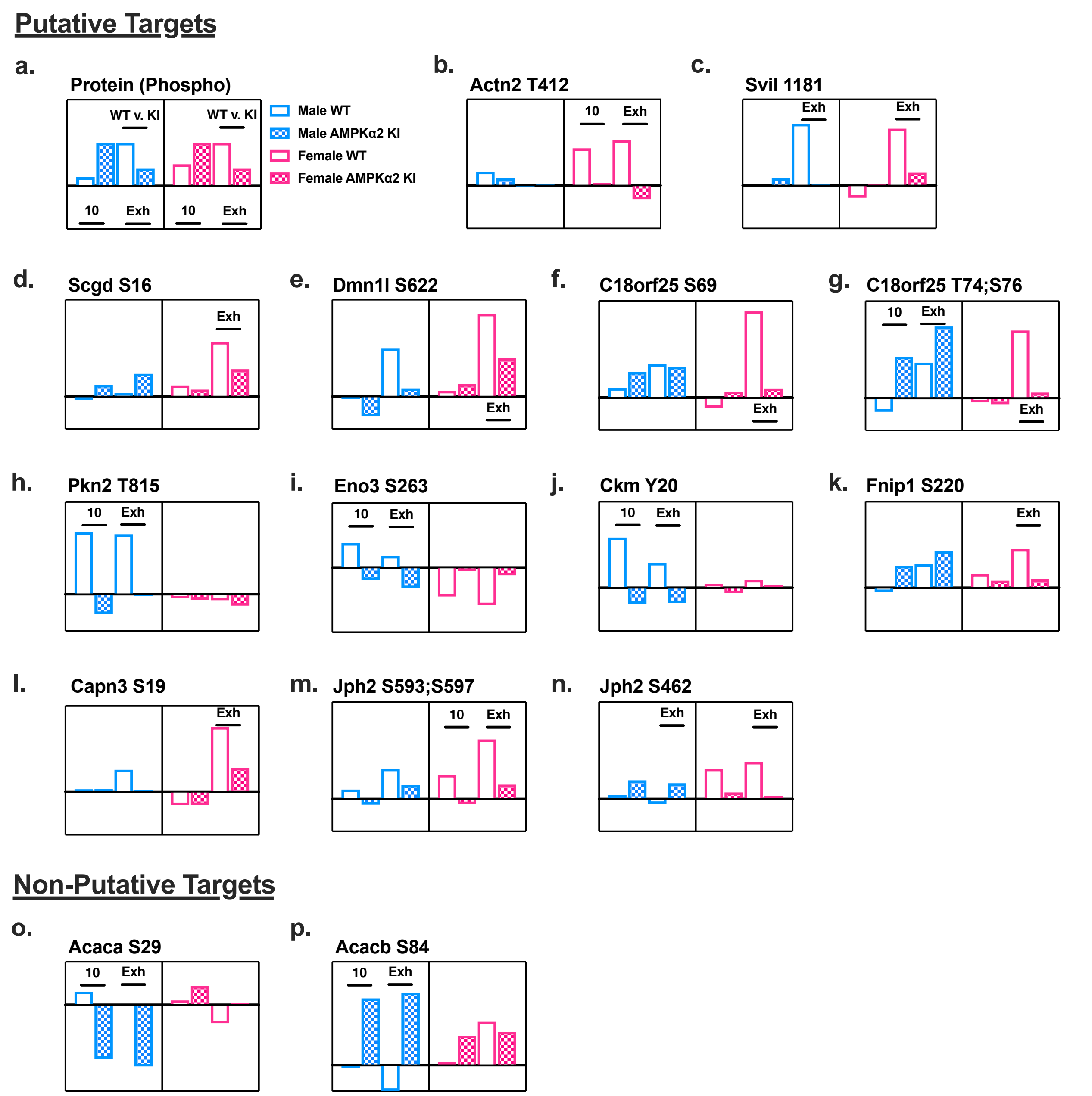

Figure S6: Select phosphoproteomic annotations from Ampkα2 T172 phosphoproteomic response to exercise within skeletal muscle.

(**A**) WT (open bar) and KI (checkered bar) mice were divided into three treatments (1) sedentary, (2) exercise for 10 minutes (10min) and (3) exercise to exhaustion (Exh) groups further by sex for male (M; blue) and female (F; pink). Bars above timepoints indicate significant differences between WT and Ampkα2 KI mice at 10 minutes or exhaustion. Complete data provided in **supplemental data file 2**. (**B-N**) Log2FC of phosphorylation sites representing significantly increased phosphorylation in males and/or female WT (adj. $p<0.05$) *and* a significant decrease compared to KI ($p<0.05$) at exhaustion. (**O-P**) Log2FC of phosphorylation sites representing significantly altered phosphorylation in males and/or female WT (adj. $p<0.05$) *or* a significant altered compared

to KI ($p<0.05$) at exhaustion. Actn2 actinin2; Svil supervilin; Scgd sarcoglycan delta; Dnml1 dynamin like protein; Pkn2 protein kinase N2; Eno3 enolase 3; Ckm creatine kinase muscle; Fnip1 follicular interacting protein 1; Capn3 calpain 3; Jph2 junctophilin 2; Acaca (ACC1) acetyl-CoA carboxylase 1; Acacb (ACC2) acetyl-CoA carboxylase 2.

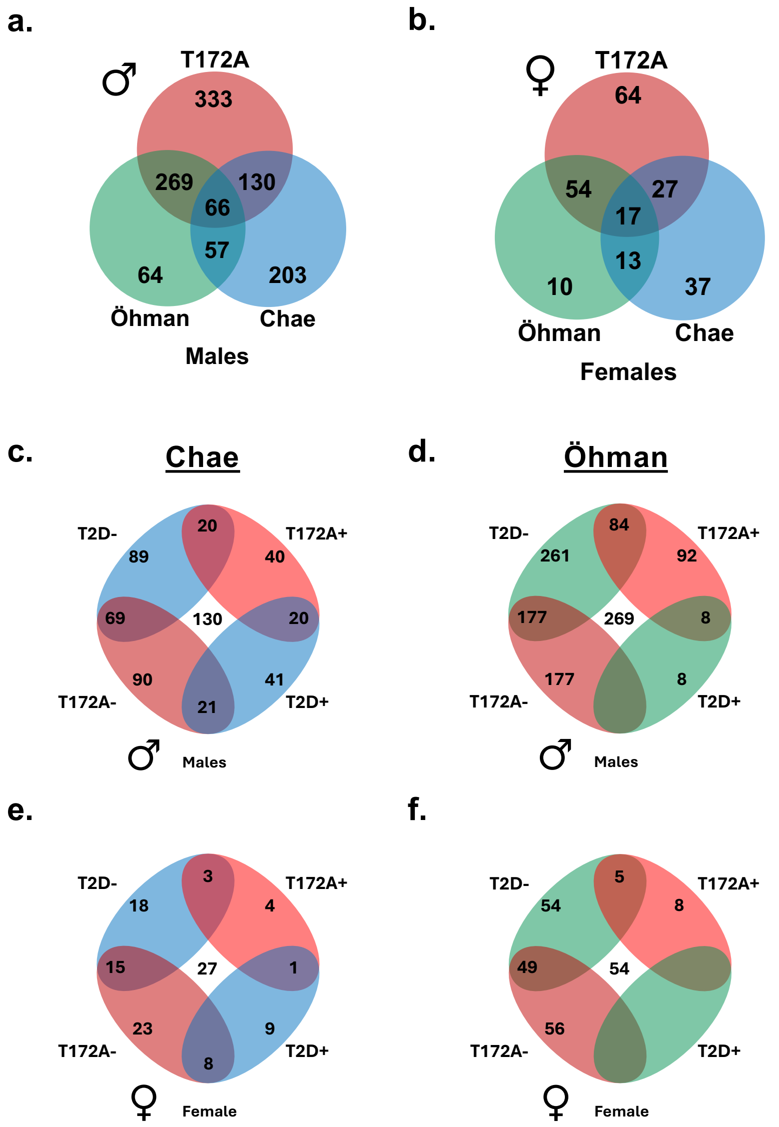

Figure S7: Supplement: Global proteomics of Ampkα2 T172A KI shares coordinate regulation with type 2 diabetic patient skeletal muscle. (**A/B**) Males (M) and females (F) from our data set (red) share 66 and 17 individual proteins respectively with both Öhman (green) and Chae (blue) the diabetic data sets. The blue and green circles that do not overlap represent the number of genes that were present in the respective studies but significantly altered in the Ampkα2 T172A data set (Chae 203M; 37F / Öhman 62M, 10F). (**C-F**) Where overlap was observed (Chae 130M; 27F/ Öhman 269M, 54F) results were distributed in the 4-way matrices dependent on agreement between positive (+) and negative (-) regulation of T172A genes and the diabetic data sets.

Supplementary Material File Captions

Supplemental Data File 1: Global Proteomics performed in gastrocnemius of sedentary wild type and Ampkα2 T172A male and female mice. Data separated by raw Z score and Correlation Adjusted MEan RAnk gene set test (CAMERA-PR) analysis.

Supplemental Data File 2: Phosphoproteomics performed in gastrocnemius of sedentary wild type and Ampkα2 T172A male and female mice, compared to 10 minutes of exercise, and exhaustive exercise.

Supplemental Data File 3: Ingenuity pathway analysis of phosphoproteomics data set comparing sedentary, 10 minutes of exercise, and exhaustive exercise.

Supplemental Data File 4: Ampk consensus matching of phosphoproteomics results with the AMPK consensus sequence for identification of putative Ampkα2 targets at strict, relaxed, and minimal match alignment.

Supplemental Data File 5: Metabolomics of gastrocnemius for sedentary wild type and Ampkα2 T172A male and female mice, compared to 10 minutes of exercise, and exhaustive exercise.
